# Shape-adaptive single-molecule magnetism and hysteresis up to 14 K in oxide clusterfullerenes Dy_2_O@C_72_ and Dy_2_O@C_74_ with fused pentagon pairs and flexible Dy–(μ_2_-O)–Dy angle†
†Electronic supplementary information (ESI) available. CCDC 1974305 and 1974314. For ESI and crystallographic data in CIF or other electronic format see DOI: 10.1039/d0sc00624f


**DOI:** 10.1039/d0sc00624f

**Published:** 2020-04-20

**Authors:** Georgios Velkos, Wei Yang, Yang-Rong Yao, Svetlana M. Sudarkova, XinYe Liu, Bernd Büchner, Stanislav M. Avdoshenko, Ning Chen, Alexey A. Popov

**Affiliations:** a Leibniz Institute for Solid State and Materials Research Helmholtzstraße 20 , 01069 Dresden , Germany . Email: s.avdoshenko@ifw-dresden.de ; Email: a.popov@ifw-dresden.de; b College of Chemistry , Chemical Engineering and Materials Science , Soochow University , Suzhou , Jiangsu 215123 , P.R. China . Email: chenning@suda.edu.cn; c Department of Chemistry , University of Texas at El Paso , 500 W University Avenue , El Paso , Texas 79968 , USA; d Chemistry Department , Moscow State University , 119991 Moscow , Russia

## Abstract

Dysprosium oxide clusterfullerenes Dy_2_O@C_s_(10528)–C_72_ and Dy_2_O@C_2_(13333)–C_74_ are synthesized and characterized by single-crystal X-ray diffraction. Carbon cages of both molecules feature two adjacent pentagon pairs. These pentalene units determine positions of endohedral Dy ions hence the shape of the Dy_2_O cluster, which is bent in Dy_2_O@C_72_ but linear in Dy_2_O@C_74_. Both compounds show slow relaxation of magnetization and magnetic hysteresis. Nearly complete cancelation of ferromagnetic dipolar and antiferromagnetic exchange Dy···Dy interactions leads to unusual magnetic properties. Dy_2_O@C_74_ exhibits zero-field quantum tunneling of magnetization and magnetic hysteresis up to 14 K, the highest temperature among Dy-clusterfullerenes.

## Abstract 2

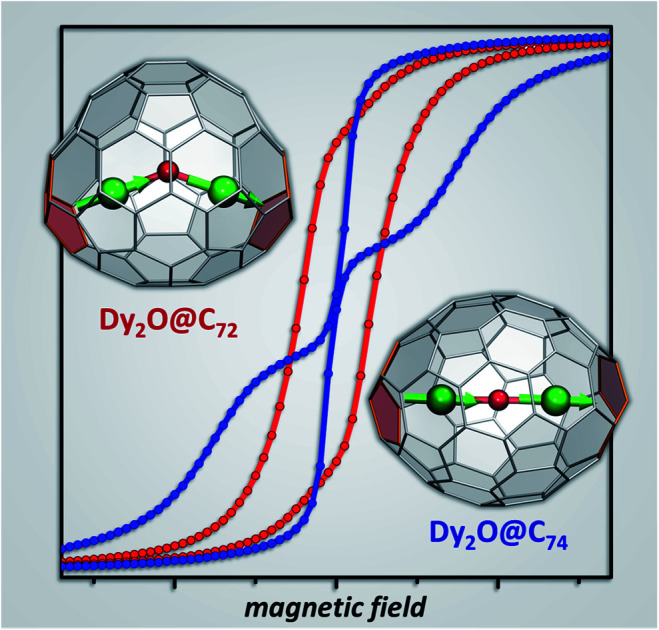
Clusterfullerenes Dy_2_O@C_72_ and Dy_2_O@C_74_ demonstrate a fine balance of exchange and dipolar interactions and slow relaxation of magnetization.

## Introduction

Endohedral metallofullerenes (EMFs) comprising metal and non-metal atoms in their endohedral species are known as clusterfullerenes.^1^ Enhanced electrostatic interactions between endohedral lanthanides (Ln) and non-metal ions (X^*q*–^), such as N^3–^, S^2–^, or O^2–^, reinforced by exceptionally short Ln–X bonds result in a strong magnetic anisotropy of Ln ions and lead to the single-molecule magnetism in many Ln-clusterfullerenes.^2^ Especially Dy-clusterfullerenes are known as robust single molecule magnets (SMMs) with many favourable properties, including thermal and chemical stability, reasonably high blocking temperatures of magnetization, and magnetic hysteresis in bulk samples as well as in monolayers on different substrates.^3^ Thus, with the improvement of EMF-SMMs, the tremendous progress achieved over the last two decades in the Ln-SMM field^4^ has a good chance to further advance to the state of device-oriented functional molecular materials.

Oxide clusterfullerenes^5^ with endohedral Ln_2_O clusters were predicted to have the largest ligand-field splitting in the whole EMF family3g,^6^ and thus are viable synthetic targets as prospective SMM candidates. Indeed, our recent study of three isomers of Dy_2_O@C_82_ revealed their unique magnetic properties.^7^ The pronounced magnetic anisotropy is combined in these molecules with antiferromagnetic (AFM) exchange interactions, which are the strongest through all dinuclear Dy complexes with non-radical bridging ligands. In this Communication, we report that the encapsulation of the Dy_2_O cluster in smaller carbon cages has a profound influence on the magnetic properties and leads to the Dy_2_O@C_74_ compound with the highest temperature of magnetic hysteresis among clusterfullerene-SMMs.

## Results and discussion

### Synthesis, molecular and electronic structure

**Fig. 1:**
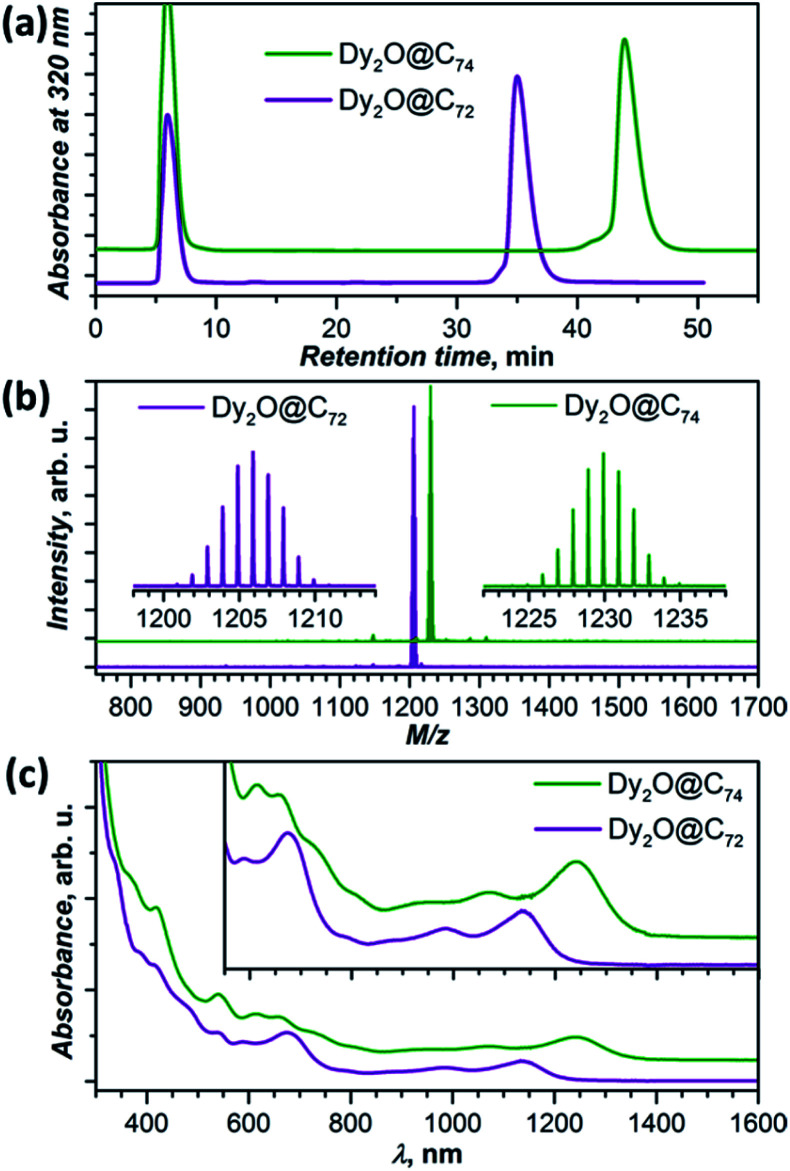
Characterization of isolated Dy_2_O@C_72_ and Dy_2_O@C_74_: (a) HPLC profiles (BuckyPrep column, toluene as eluent, flow rate 4 mL min^–1^; solvent peak appears at 6 min); (b) LDI mass-spectra in a positive-ion mode; (c) UV-Vis-NIR absorption spectra in toluene (the inset shows magnification of the low-energy range).

New Dy-oxide clusterfullerenes Dy_2_O@C_72_ and Dy_2_O@C_74_ were synthesized by arc-discharge evaporation of Dy_2_O_3_-filled graphite rods in He/CO_2_ atmosphere (200/20 Torr) and isolated by multi-step high-performance liquid chromatography (HPLC) as described in ESI.† Basic characterization of the purified Dy_2_O@C_72_ and Dy_2_O@C_74_, including their HPLC profiles, high-resolution laser desorption ionization mass spectra with characteristic isotopic patterns, and UV-vis-NIR absorption spectra are surveyed in Fig. 1.

**Fig. 2:**
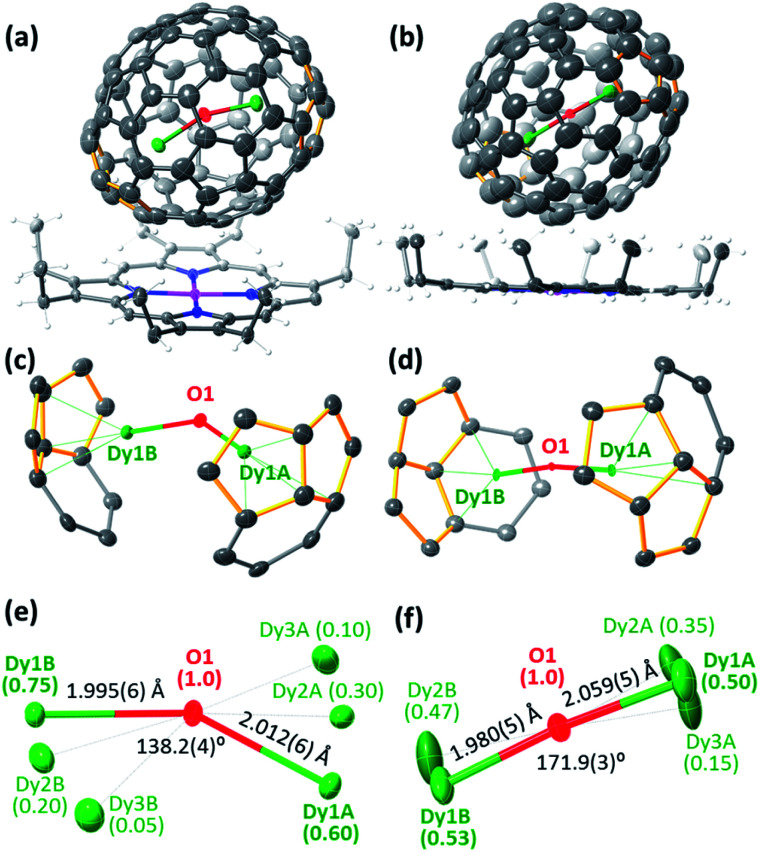
(a and b) Thermal ellipsoids of Dy_2_O@C_2*n*_·Ni^II^(OEP) crystals with 30% probability showing only the major dysprosium sites: (a) Dy_2_O@C_s_(10528)–C_72_, (b) Dy_2_O@C_2_(13333)–C_74_; the solvent molecules and minor Dy sites are omitted, Dy is green, O is red, Ni is purple, N is blue, C is grey, adjacent-pentagon pairs are highlighted in orange. (c and d) Interaction of the major Dy_2_O site with the closest cage motif in (c) Dy_2_O@C_72_, and (d) Dy_2_O@C_74_. (e and f) Endohedral Dy_2_O units with disordered Dy sites and selected structural parameters in (e) Dy_2_O@C_72_, and (f) Dy_2_O@C_74_.

The molecular structures of Dy_2_O@C_72_ and Dy_2_O@C_74_ were elucidated by single-crystal X-ray diffraction of co-crystals with Ni^II^(OEP) revealing C_*s*_(10528)–C_72_ and C_2_(13333)–C_74_ carbon cages (Fig. 2a and b).§
§Crystals were grown by layering the benzene solution of nickel octaethylporphyrin (Ni(OEP)) onto the CS_2_ solution of the Dy_2_O@C_2*n*_ (2*n* = 72, 74) isomers. The as-prepared crystals Dy_2_O@C_*s*_(10528)–C_72_·Ni(OEP)·2(C_6_H_6_) and Dy_2_O@C_2_(13333)–C_74_·Ni(OEP)·C_6_H_6_·CS_2_ were measured with Bruker APEX II at 120 and 173 K, respectively. The structures were solved using direct methods^17^ and refined on F2 using full-matrix least-squares using the SHELXL2015 crystallographic software package.^18^ Hydrogen atoms were inserted at calculated positions and constrained with isotropic thermal parameters. The crystal data are presented in Table S1.† The data can be obtained free of charge from the Cambridge Crystallographic Data Centre with CCDC no. 1974305 and 1974314,† respectively. Both fullerenes violate the Isolated Pentagon Rule (IPR) and have two pairs of adjacent pentagons, which are stabilized by coordination with Dy ions (Fig. 2c,d). The C_*s*_(10528)–C_72_ cage was found before in Sc_2_S@C_72_,^8^ Sc_2_C_2_@C_72_,^9^ and presumably Dy_2_S@C_72_.3*g* The C_2_(13333)–C_74_ cage was predicted theoretically as a plausible isomer of Sc_2_C_2_@C_74_ 10 and was found recently in Ho_2_O@C_74_.5*c*

Carbon cages and oxygen atoms are fully ordered in the crystals, but Dy atoms are less ordered with 2–3 sites for each metal atom (Fig. 2e and f). In Dy_2_O@C_72_ the Dy–O–Dy angle is 138.2(4)° for the major Dy sites (occupancy 0.60 and 0.75), whereas in Dy_2_O@C_74_ the Dy_2_O cluster is close to the linear shape, similar to the Ho_2_O cluster in Ho_2_O@C_2_(13333)–C_74_.5*c* Dy–O bonds are very short, 1.980(5)–2.059(5) Å, but the disorder in Dy positions does not allow detailed analysis. Optimization of the molecular structures performed at the PBE-D level (PAW 4f-in-core potentials, VASP 5.0 code^11^) resulted in the Dy–O distances and Dy–O–Dy angles of 2.025 Å/138° in Dy_2_O@C_72_ and 2.038 Å/180° in Dy_2_O@C_74_.

Apparently, the variation of the Dy_2_O cluster shape from bent to linear is dictated by the Dy···Dy distance, which changes from 3.743(1) Å in Dy_2_O@C_72_ to 4.030(2) Å in Dy_2_O@C_74_ (DFT values are 3.784 and 4.076 Å, respectively). In due turn, the location of Dy atoms inside the fullerene is determined by the arrangement of pentalene units. The distance between centroids of pentalenes in X-ray structures is 8.151 Å in Dy_2_O@C_72_ and 8.597 Å in Dy_2_O@C_74_. Thus, the shape of the Dy_2_O cluster is imposed by the fullerene cage form-factor.1*c* Note that in Dy_2_O@C_82_ isomers the cluster is bent despite the larger cage size than in Dy_2_O@C_74_: in fact, Dy···Dy distances in all Dy_2_O@C_82_ structures are shorter than in Dy_2_O@C_74_.^7^ At the same time, the linear shape of the cluster was found in Ho_2_O@*D*_2d_(23)–C_84_, which also has an elongated shape of the fullerene cage.5*d*

DFT-based molecular dynamics simulations showed that at 300 K on a timescale of 100 ps metal atoms oscillate near their optimized positions (Fig. S4†). IR spectra calculated from the Fourier transform of the time-dependent dipole moment resemble those computed for the static model and agree well with the experimental spectra (Fig. S5†). Antisymmetric Dy–O stretching vibration is found at 680 cm^–1^ in Dy_2_O@C_72_ and 700 cm^–1^ in Dy_2_O@C_74_.

Electronic absorption spectra of both compounds extend to the near-IR region with the lowest-energy bands at 1137 nm (1.09 eV) in Dy_2_O@C_72_ and 1240 nm (1.00 eV) in Dy_2_O@C_74_ (Fig. 1c). The spectrum of Dy_2_O@C_72_ exhibits a close similarity to the spectra of Sc_2_S@C_72_,^8^ Sc_2_C_2_@C_72_,^9^ and Dy_2_S@C_72_3*g* featuring the same C_*s*_(10528)–C_72_ cage. However, the cluster composition has a noticeable impact on the lowest energy excitation, found at 1076 nm (Sc_2_S), 1082 nm (Sc_2_C_2_), or 1115 nm (Dy_2_S). The absorption spectrum of Dy_2_O@C_74_ is virtually identical to that of Ho_2_O@C_2_(13333)–C_74_.5*c*

Further insight into the electronic structure of Dy_2_O clusterfullerenes is obtained from the electrochemical studies. Cyclic voltammograms are shown in Fig. S6† and redox potentials are listed in Table 1. Both Dy_2_O@C_72_ and Dy_2_O@C_74_ exhibit one reversible oxidation and three reversible reduction steps as well as poorly reversible fourth reduction and second oxidation steps. DFT calculations show that frontier molecular orbitals of both molecules are localized on their fullerene cages (Fig. S7†), which suggests that the redox processes do not affect the Dy_2_O cluster. Comparison of the redox potentials of Dy_2_O@C_72_ to Sc_2_S@C_72_ 8 and Sc_2_C_2_@C_72_ 9 (Table S2†) reveals that the variation of the optical gap discussed above is also reflected in their electrochemical gaps despite the predominant localization of the frontier MOs on the carbon cage in all molecules.

**Table 1:** Redox potentials^*a*^ of Dy_2_O@C_*s*_(10528)–C_72_ and Dy_2_O@C_2_(13333)–C_74_

EMF	O–II	O–I	R–I	R–II	R–III	R–IV	Gap_EC_
Dy_2_O@C_72_	0.87	0.33	–1.09	–1.56	–2.18	–2.55	1.42
Dy_2_O@C_74_	1.18	0.52	–0.81	–1.17	–2.16	–2.56	1.33

^*a*^Measured in TBAPF_6_/*o*-dichlorobenzene and referred *versus* Fe(Cp)_2_^+/0^.

### Magnetic properties

#### SQUID magnetometry

**Fig. 3:**
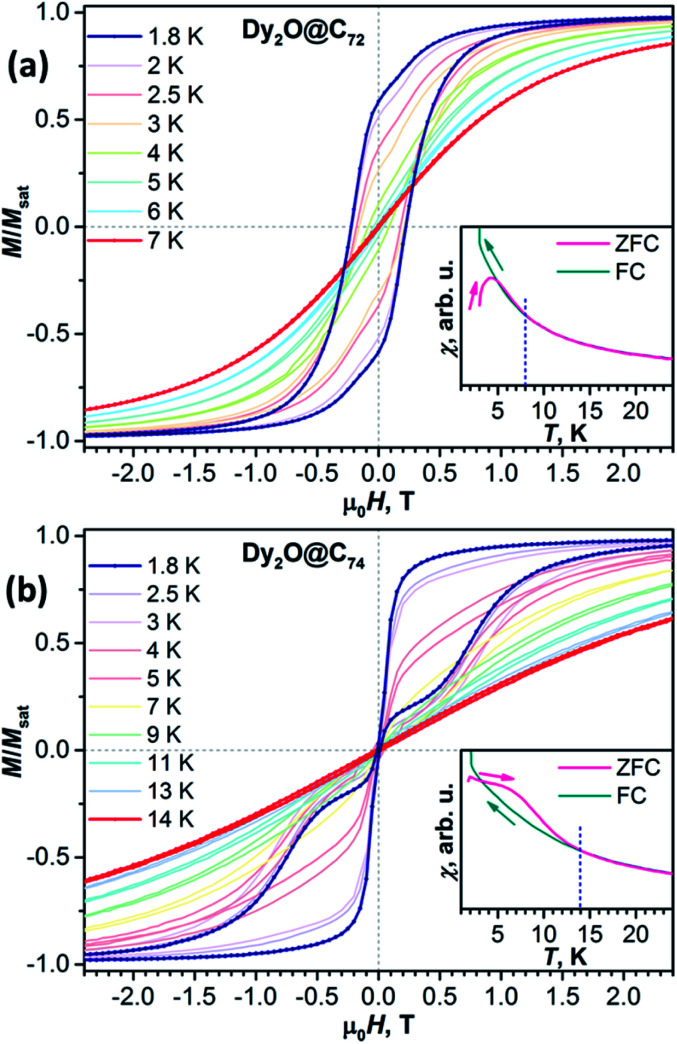
Magnetic hysteresis curves of Dy_2_O@C_72_ (a) and Dy_2_O@C_74_ (b) measured with the sweep rate 2.9 mT s^–1^. Insets show comparison of *χ*_FC_ and *χ*_ZFC_ curves (*μ*_0_*H* = 0.2 T, temperature sweep rate 5 K min^–1^); dashed blue lines denote *T*_irrev_ as the temperature, at which *χ*_FC_ and *χ*_ZFC_ curves bifurcate.

Magnetic properties of Dy_2_O@C_72_ and Dy_2_O@C_74_ powders were studied by SQUID magnetometry. Fig. 3a and b show low-temperature magnetization curves measured for two clusterfullerenes with a sweep rate of 2.9 mT s^–1^. Dy_2_O@C_72_ shows open hysteresis up to 7 K (Fig. S8†). In the isostructural Dy_2_S@C_72_, the hysteresis is much narrower and is closing already at 3 K.3*g* Thus, once again Dy_2_O cluster is found to exhibit more robust single molecule magnetism than the Dy_2_S analog with the same fullerene cage. Earlier, superior SMM properties were found for Dy_2_O@C_82_ when compared to the isostructural Dy_2_S@C_82_.^7^ At the same time, the use of softer sulfur co-ligands was found to be beneficial to increase the axiality of other types of single-molecule magnets.^12^

Magnetic hysteresis in Dy_2_O@C_74_ closes above 14 K (Fig. S8†). The shape of the hysteresis with the abrupt decay of magnetization near 0 T is different from that of Dy_2_O@C_72_ and other dinuclear Dy-clusterfullerenes. Such a “waist-restricted” or “butterfly” hysteresis is characteristic for the quantum tunnelling of magnetization (QTM) in zero field and is common for single-ion magnets, but is not typical for dinuclear SMMs.

Blocking of magnetization is analysed by comparing magnetic susceptibility measured after cooling the sample in zero field (*χ*_ZFC_) and during cooling the sample in field (*χ*_FC_).^13^ Blocking temperature *T*_B_ defined as the peak temperature of *χ*_ZFC_ is near 4 K in Dy_2_O@C_72_ and 6.7 K in Dy_2_O@C_74_ (Fig. 3) when measured in a field of 0.2 T with the temperature sweep rate of 5 K min^–1^. However, both fullerenes show bifurcation of *χ*_ZFC_ and *χ*_FC_ curves (defined as *T*_irrev_^13^) until noticeably higher temperatures, reaching 8 K for Dy_2_O@C_72_ and 14 K for Dy_2_O@C_74_. The uncommon shape of *χ*_ZFC_, being higher than *χ*_FC_, is caused in Dy_2_O@C_74_ by the fast QTM at small fields. It should be also noted that *T*_B_ and *T*_irrev_ values are kinetic parameters and depend on the magnetic field, sweep rate, and some technical aspects of the measurements (see Fig. S9–S11† for further measurements and ref. 4d for a more detailed discussion). More universal parameter is the temperature *T*_B100_, at which relaxation time of magnetization is 100 s. As determined from the relaxation time measurements described below, *T*_B100_ of Dy_2_O@C_72_ is 3.4 K in zero field and 2.6 K at 0.2 T. For Dy_2_O@C_74_, the *T*_B100_ value in zero field is not defined because QTM limits the relaxation time, whereas in the field of 0.2 T the value is 5.0 K.

Relaxation times of magnetization *τ*_M_ below *T*_irrev_ (Tables S3–S8 and Fig. S12†) were determined by the stretched exponential fitting of magnetization decay curves recorded after the fast sweep of the magnetic field from 5 T to a required value. Unfortunately, the isolable amounts of the clusterfullerenes are insufficient for the measurement of relaxation times at higher temperatures by AC magnetometry.

**Fig. 4:**
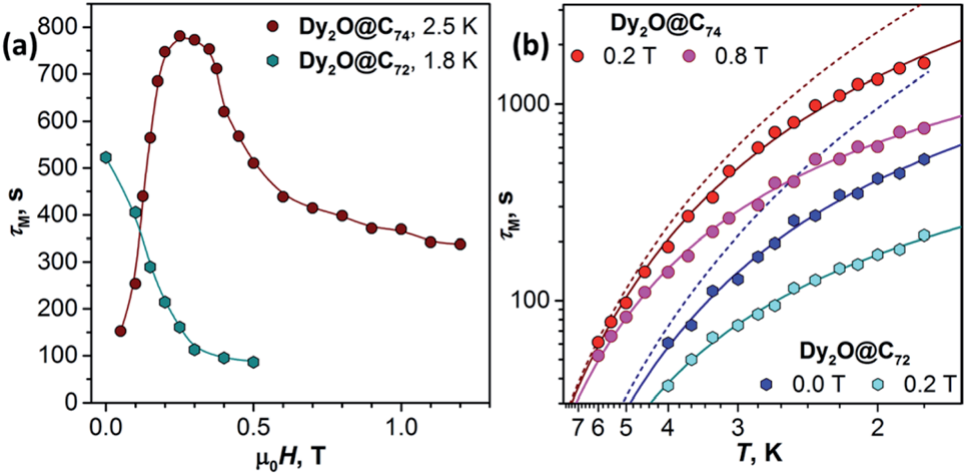
Magnetization relaxation times of Dy_2_O@C_72_ and Dy_2_O@C_74_: (a) Field dependence of *τ*_M_ at 1.8 K (Dy_2_O@C_72_) and 2.5 K (Dy_2_O@C_74_). Note that the smallest field for Dy_2_O@C_74_ is 0.05 T since the measurements in zero field are not possible because of the fast QTM process; (b) Temperature dependence of *τ*_M_ in two different fields, dots are measured values, solid lines are fits by a combination of Raman and direct mechanisms (eqn (1)), dashed lines are contributions of Raman process for Dy_2_O@C_72_ (blue) and Dy_2_O@_74_ (red).

Fig. 4a shows the magnetic field dependence of *τ*_M_ measured at a constant temperature. For Dy_2_O@C_72_, *τ*_M_ decays fast with the field from 523 s at zero field to 95 s at 0.4 T and then tends to level off. This *τ*_M_(*H*) dependence is a clear manifestation of the direct relaxation mechanism. For Dy_2_O@C_74_, relaxation rate in 0 T could not be measured because of the fast QTM, and the lowest field studied is 0.05 T. Field dependence at 2.5 K first shows a fast increase of *τ*_M_ from 152 s at 0.05 T to 750–780 s at 0.2–0.35 T. This *τ*_M_(*H*) dependence corresponds to the gradual quenching of the QTM by increasing Zeeman splitting. With the further field increase beyond 0.35 T, relaxation accelerates due to the contribution of the direct mechanism as in Dy_2_O@C_72_.

The temperature dependence of *τ*_M_ shown in Fig. 4b cannot be described by a direct process alone. Equally good fits are obtained either for a combination of the direct and Raman mechanisms:

1 *τ*_M_^–1^(*T*) = *C*_d,*H*_*T*^*n*_d_^ + *C*_R_*T*^*n*_R_^

or by a combination of the direct and Arrhenius process:

2 *τ*_M_^–1^(*T*) = *C*_d,*H*_*T*^*n*_d_^ + *τ*_0_^–1^exp(–*U*^eff^/*T*)

For the direct mechanism *n*_d_ should be 1, and *C*_d,*H*_ is field-dependent. For each fullerene, temperature dependence was measured in two different fields (Fig. 4b), and then two datasets were fitted jointly either with eqn (1) or with eqn (2) keeping field-independent parameters identical (Fig. S13 and S14†). Eqn (1) gives more physically sound interpretation of the relaxation of magnetization and will be discussed further. For Dy_2_O@C_72_, the procedure returned *n*_d_ = 1.43 ± 0.13, *C*_d,0 T_ = (5.2 ± 0.1)·10^–4^ s^–1^ K^–1.4^, *C*_d,0.2 T_ = (1.8 ± 0.2)·10^–3^ s^–1^ K^–1.4^, *n*_R_ = 3.7 ± 0.4, and *C*_R_ = (8 ± 4)·10^–5^ s^–1^ K^–3.7^. The *n*_d_ value is higher than 1, which may be due to the phonon bottleneck effect (which increases *n*_d_ to 2). For Dy_2_O@C_74_, we obtained *n*_d_ = 1.23 ± 0.14, *C*_d,0.2 T_ = (1.3 ± 0.3)·10^–4^ s^–1^ K^–1.25^, *C*_d,0.8 T_ = (4.9 ± 0.5)·10^–4^ s^–1^ K^–1.25^, *n*_R_ = 3.28 ± 0.14, and *C*_R_ = (4 ± 1)·10^–5^ s^–1^ K^–3.3^. For both molecules the direct mechanism dominates at lower temperature and at higher fields, whereas the field-independent Raman process with *n*_R_ of 3–4 takes over at higher temperatures. These relatively small *n*_R_ values indicate that optical phonons contribute strongly to the relaxation of magnetization.^14^ Contribution of the Raman mechanism is shown for each molecule in Fig. 4b. When eqn (2) was used for the fit, Arrhenius processes with effective barriers of 14–16 K were obtained (see ESI†). These values are much smaller than the ligand-field splitting of Dy^3+^ ions in Dy_2_O@C_2*n*_ molecules, but are considerably larger than the energy difference between the states with ferromagnetic and antiferromagnetic coupling of magnetic moments (see below), and thus cannot match the spin energy levels of the system.

#### Dy···Dy interactions and magnetic anisotropy

To get better understanding of the Dy···Dy coupling, magnetization curves of Dy_2_O@C_72,74_ molecules were simulated and fitted using powder averaging in the PHI code^15^ with the following spin Hamiltonian:3
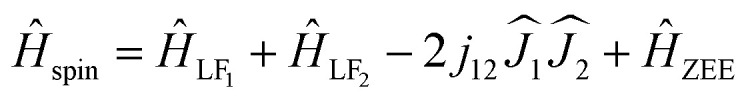
where *Ĥ*_LF_*i*__ are single-ion ligand-field Hamiltonians of Dy^3+^ with *ab initio* computed parameters, *j*_12_ is the coupling constant between dysprosium moments, and *Ĥ*_ZEE_ is Zeeman term. Dy^3+^ moments 
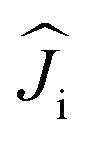
 are treated in the |*J*,*m*_*J*_〉 basis sets ( basis sets (^6^*H*_15/2_ multiplet). In this notation, the energy difference between FM and AFM-coupled states of the molecule is Δ*E*_AFM–FM_ = 225*j*_12_ cos(*α*), where *α* is the angle between quantization axes of two Dy^3+^ ions.

**Fig. 5:**
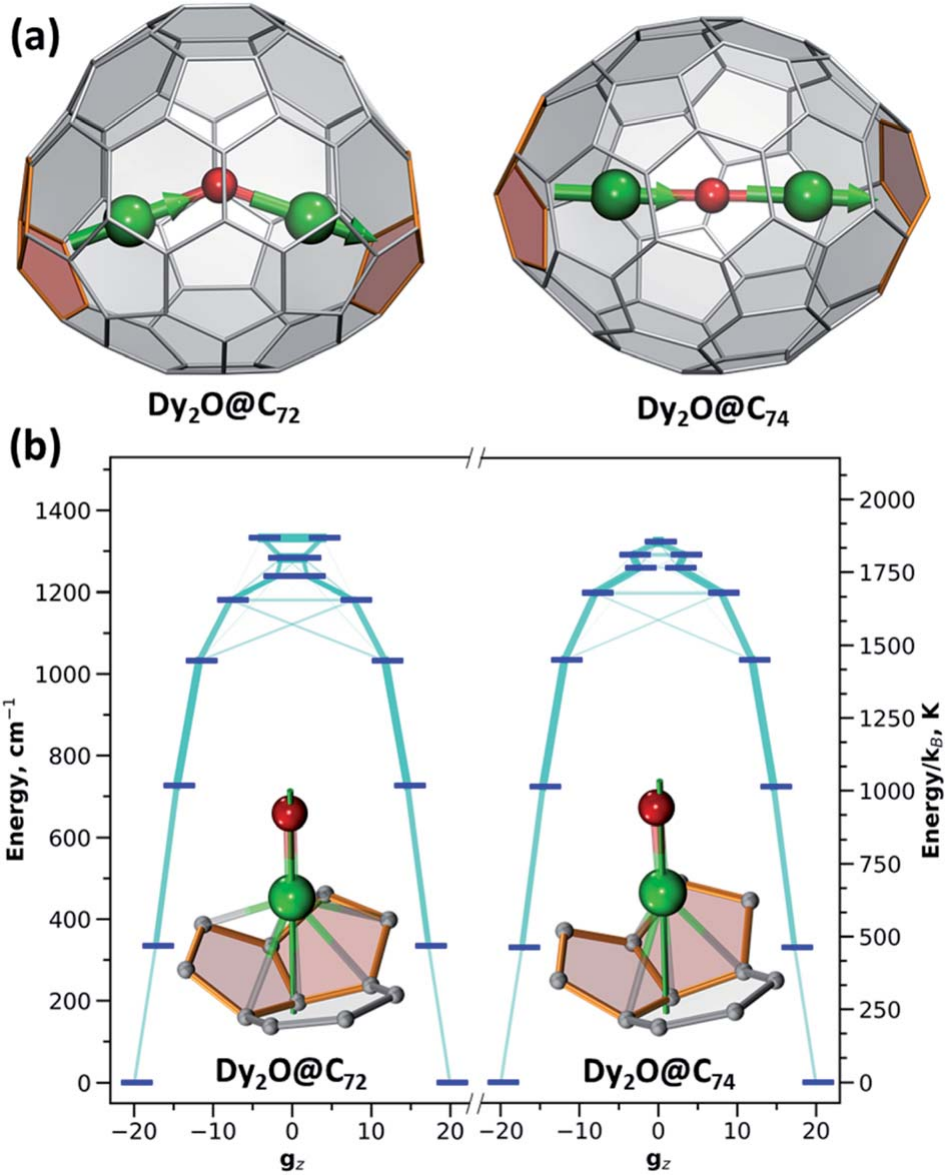
(a) DFT-optimized molecular structures of Dy_2_O@C_72_ and Dy_2_O@C_74_ showing alignment of magnetic moment in ferromagnetically-coupled ground-state doublet (Dy is green, O is red, adjacent pentagon pairs are rose; magnetic moments of Dy ions are shown as green arrows). (b) CASSCF/RASSI-computed ligand-field splitting for Dy^3+^ ions in Dy_2_O@C_72_ and Dy_2_O@C_74_; the thickness of light blue lines corresponds to transition probability. The insets in (b) show Dy-coordinated fragments of the fullerene cage and quantization axis of Dy (green line); Dy–C distances shorter than 2.6 Å are shown as bonds. The scale on the left shows the energy in cm^–1^, on the right in K.

The ligand-field parameters for Dy ions were computed *ab initio* at the CASSCF(9,7)/SO-RASSI^16^ level for DyYO@C_72_ and DyYO@C_74_ molecules. Given the considerable disorder of experimental structures, DFT-optimized atomic coordinates were used. *Ab initio* calculations showed that Dy^3+^ ions in both clusterfullerenes have easy-axis magnetic anisotropy with the quantization axes aligned along the corresponding Dy–O bonds with a deviation of 2°. In |*J*,*m*_*J*_〉 representation, the four lowest energy Kramers doublets (KDs) are almost pure states with representation, the four lowest energy Kramers doublets (KDs) are almost pure states with *m*_*J*_ of 15/2 (ground state), 13/2 (near 340 cm^–1^), 11/2 (near 720 cm^–1^), and 9/2 (near 1030 cm^–1^) (Table S9†). Transition probabilities between these states are thus very low (Fig. 5). Further KDs have a more mixed nature, and at higher temperatures the relaxation of magnetization following the Orbach mechanism is expected to proceed *via* the fifth KD at 1180–1200 cm^–1^, which resembles the situation found experimentally in Dy_2_ScN@C_80_ with the Orbach barrier of 1206 ± 15 cm^–1^.3*e* The overall LF splitting is 1337 cm^–1^ in Dy_2_O@C_72_ and 1329 cm^–1^ in Dy_2_O@C_74_. In brief, both clusterfullerenes have strongly axial magnetic anisotropy with similar energies and state compositions, and their low-temperature magnetic properties are determined exclusively by the ground state KDs.

The coupling constants *j*_12_ defining the scale of Dy···Dy interactions are determined by fitting of the experimental magnetization curves to eqn (3) (Fig. S15 and S16†). For Dy_2_O@C_72_, the best fit is obtained for the ferromagnetic coupling with *j*_12_ = 0.009 ± 0.002 cm^–1^, which gives Δ*E*_AFM–FM_ = 1.5 ± 0.3 cm^–1^. For Dy_2_O@C_74_, the optimal *j*_12_ is less than 0.001 cm^–1^ with the uncertainty of ±0.002 cm^–1^. The FM and AFM states in Dy_2_O@C_74_ are thus degenerate within |Δ*E*_AFM–FM_| < 0.4 cm^–1^, which means that Dy^3+^ moments are essentially decoupled.

If a magnetic moment of one of the centers in a dinuclear FM-coupled system is flipped, the system arrives in the AFM-coupled state (and *vice versa*). As long as the FM and AFM states have a different energy, the Δ*E*_AFM–FM_difference acts as a barrier preventing the QTM, because the latter requires degeneracy of the energy levels. In all dinuclear Dy-clusterfullerenes studied to date the Δ*E*_AFM–FM_ difference, either positive (FM) or negative (AFM), was large enough to prevent the QTM in zero field. Dy_2_O@C_74_ is the first dinuclear Dy-clusterfullerene exhibiting efficient zero-field QTM, which can be explained by the vanishing Dy···Dy coupling. In Dy_2_O@C_72_, Dy···Dy coupling is also weak but still sufficient to quench the QTM in zero field.

The Dy···Dy coupling in the Dy_2_O clusters found in this work – weak FM in Dy_2_O@C_72_ and negligible in Dy_2_O@C_74_ – is in a sharp contrast with the situation in Dy_2_O@C_82_ isomers, featuring strong AFM coupling with Δ*E*_AFM–FM_ of (5.4–12.9) cm^–1^.^7^ Such a large variation of the strength of magnetic interactions in seemingly very similar molecules requires a closer look. The overall Dy···Dy coupling in dinuclear spin systems can be divided into exchange and dipolar contributions, Δ*E*exchAFM–FM and Δ*E*dipAFM–FM. The latter can be computed exactly when molecular structures and orientations of magnetic moments are known. Using DFT-optimized structures and orientation of quantization axes from *ab initio* calculations, we obtain Δ*E*dipAFM–FM of 2.99 cm^–1^ in Dy_2_O@C_72_ and 2.56 cm^–1^ in Dy_2_O@C_74_. Thus, dipolar interactions favour FM arrangement of Dy^3+^ moments and are of the same size as in Dy_2_O@C_82_ isomers with Δ*E*dipAFM–FM of 2.5–3.0 cm^–1^. Apparently, small overall coupling in Dy_2_O@C_72,74_ results from the cancellation of dipolar coupling by exchange interactions, which are therefore antiferromagnetic. To yield the experimentally determined Δ*E*_AFM–FM_ energies, Δ*E*exchAFM–FM values should be –1.5 ± 0.3 cm^–1^ in Dy_2_O@C_72_ and –2.5 ± 0.4 cm^–1^ in Dy_2_O@C_74_. Thus, it is the antiferromagnetic exchange coupling in the Dy_2_O cluster that is changing strongly from one fullerene to another and is therefore responsible for the considerable variation of the magnetic properties in the series of Dy_2_O clusterfullerenes. The factors determining exchange interactions in Dy-clusterfullerenes are not very clear yet, and further studies of Dy-oxide clusterfullerenes with different fullerene cages are needed to establish structure–property correlations.

## Conclusions

In this work we isolated Dy_2_O@C_*s*_(10528)–C_72_ and Dy_2_O@C_2_(13333)–C_74_ clusterfullerenes violating the isolated pentagon rule and characterized their structural, electronic, and magnetic properties. The shape of the Dy_2_O cluster is determined by the form-factor of the carbon cage and location of pentalene units, leading to the bent cluster in Dy_2_O@C_72_ but linear one in Dy_2_O@C_74_. Both fullerenes are single molecule magnets showing hysteresis up to 7 K in Dy_2_O@C_72_ and 14 K in Dy_2_O@C_74_, the latter being the highest temperature among Dy-clusterfullerenes. The magnetic Dy···Dy interactions in Dy_2_O clusters are characterized by the ferromagnetic dipolar coupling counterbalanced by the antiferromagnetic exchange. In Dy_2_O@C_74_, these two contributions cancel each other completely leading to decoupled Dy moments and zero-field quantum tunnelling of magnetization. As a result, magnetization of Dy_2_O@C_74_ relaxes fast in zero field, but the relaxation becomes slow once the tunnelling is quenched in a finite magnetic field. This study demonstrates that the shape flexibility of the Dy_2_O cluster and its conformity with the fullerene form-factor is reflected in the substantial variation of the Dy_2_O-clusterfullerene magnetic properties with the carbon cage. This cage-adaptive SMM behaviour suggests that even stronger variability of magnetic properties may be achieved in future exploration of lanthanide-oxide clusterfullerenes with different carbon cages.

## Conflicts of interest

There are no conflicts to declare.

## Supplementary Material

Supplementary information

Crystal structure data

## References

[cit1] (b) Popov A. A., Endohedral Fullerenes: Electron Transfer and Spin, Springer International Publishing, Cham, 2017.

[cit2] Liu F., Wang S., Gao C.-L., Deng Q., Zhu X., Kostanyan A., Westerström R., Jin F., Xie S.-Y., Popov A. A., Greber T., Yang S. (2017). Angew. Chem., Int. Ed..

[cit3] Spree L., Popov A. A. (2019). Dalton Trans..

[cit4] Harriman K. L. M., Errulat D., Murugesu M. (2019). Trends in Chemistry.

[cit5] Abella L., Wang Y., Rodríguez-Fortea A., Chen N., Poblet J. M. (2017). Inorg. Chim. Acta.

[cit6] Singh M. K., Rajaraman G. (2016). Chem. Commun..

[cit7] Yang W., Velkos G., Liu F., Sudarkova S. M., Wang Y., Zhuang J., Zhang H., Li X., Zhang X., Büchner B., Avdoshenko S. M., Popov A. A. (2019). Adv. Sci..

[cit8] Chen N., Beavers C. M., Mulet-Gas M., Rodriguez-Fortea A., Munoz E. J., Li Y.-Y., Olmstead M. M., Balch A. L., Poblet J. M., Echegoyen L. (2012). J. Am. Chem. Soc..

[cit9] Feng Y., Wang T., Wu J., Feng L., Xiang J., Ma Y., Zhang Z., Jiang L., Shu C., Wang C. (2013). Nanoscale.

[cit10] Zhao P., Zhao X., Ehara M. (2017). Inorg. Chem..

[cit11] Hafner J. (2008). J. Comput. Chem..

[cit12] Murrie M., Canaj A., Dey S., Cespedes O., Wilson C., Rajaraman G. (2020). Chem. Commun..

[cit13] Gatteschi D., Sessoli R. and Villain J., Molecular Nanomagnets, Oxford University Press, New York, 2006.

[cit14] Singh A., Shrivastava K. N. (1979). Phys. Status Solidi B.

[cit15] Chilton N. F., Anderson R. P., Turner L. D., Soncini A., Murray K. S. (2013). J. Comput. Chem..

[cit16] Aquilante F., Autschbach J., Carlson R. K., Chibotaru L. F., Delcey M. G., De Vico L., Galván I. F., Ferré N., Frutos L. M., Gagliardi L., Garavelli M., Giussani A. (2016). J. Comput. Chem..

[cit17] Dolomanov O. V., Bourhis L. J., Gildea R. J., Howard J. A., Puschmann H. (2009). J. Appl. Crystallogr..

[cit18] Sheldrick G. M. (2015). Acta Crystallogr., Sect. C: Struct. Chem..

