# Supplementary material for: Shape-adaptive single-molecule magnetism and hysteresis up to 14 K in oxide clusterfullerenes Dy2O@C72 and Dy2O@C74 with fused pentagon pairs and flexible Dy–(μ2-O)–Dy angle
Source: Chem Sci. 2020 Apr 20;11(18):4766–72. doi: 10.1039/d0sc00624f (PMC7116574; doi:10.1039/d0sc00624f)
Supplement: Supplementary file 1 [file SC-011-D0SC00624F-s001.pdf]

Electronic Supplementary Material (ESI) for Chemical Science.
This journal is © The Royal Society of Chemistry 2020

Shape-adaptive single-molecule magnetism and hysteresis up to 14 K in oxide clusterfullerenes $Dy_2O@C_{72}$ and $Dy_2O@C_{74}$ with fused pentagon pairs and flexible Dy–(μ_2-O)–Dy angle

Georgios Velkos,[#a] Wei Yang,[#b] Yang-Rong Yao,[c] Svetlana M. Sudarkova,[a,d] XinYe Liu,[b] Bernd Büchner,[a] Stanislav M. Avdoshenko,*[a] Ning Chen*[b] and Alexey A. Popov*[a]

Supporting information

Synthesis and separation	S2
Single-crystal X-ray analysis	S4
DFT-based Molecular Dynamics	S5
IR Spectroscopy	S6
Electrochemical properties	S7
Frontier Molecular Orbitals	S8
Measurements of magnetic properties	S9
ZFC and FC measurements	S10
Magnetization relaxation times	S13
Ab initio calculations of ligand-field splitting	S19
Fitting of magnetization curves	S20
Experimental and simulated (M/H)T curves	S21
DFT-optimized Cartesian coordinates	S22
References	S26

Synthesis and separation

$Dy_2O@C_s(10528)$-C_{72} and $Dy_2O@C_2(13333)$-C_{74} were synthesized by a modified Krätschmer-Huffman DC arc-discharge method. The carbon rod filled with 1.27 g of Dy2O3 powder and 2.3 g of graphite powder (molar ratio of Dy/C = 1:24) was vaporized under a He/CO_2 atmosphere (200 Torr of helium with 20 Torr of CO_2 added). The soot was collected and refluxed in carbon disulfide (CS_2) under an argon atmosphere for 12 h. Totally, 200 rods were vaporized and ca. 4.0 g crude fullerene extract was obtained (ca. 20mg per rod). The crude extract was treated with $TiCl_4$, which removed most of the empty fullerenes (Fig. S1). $Dy_2O@C_{72}$ and $Dy_2O@C_{74}$ were isolated and purified by multistage high-performance liquid chromatography (HPLC) as shown in Fig. S2 and S3, giving ca. 0.8 mg $Dy_2O@Cs(10528)$-C_{72} and 0.6 mg $Dy_2O@C_2(13333)$-C_{74} in the end.

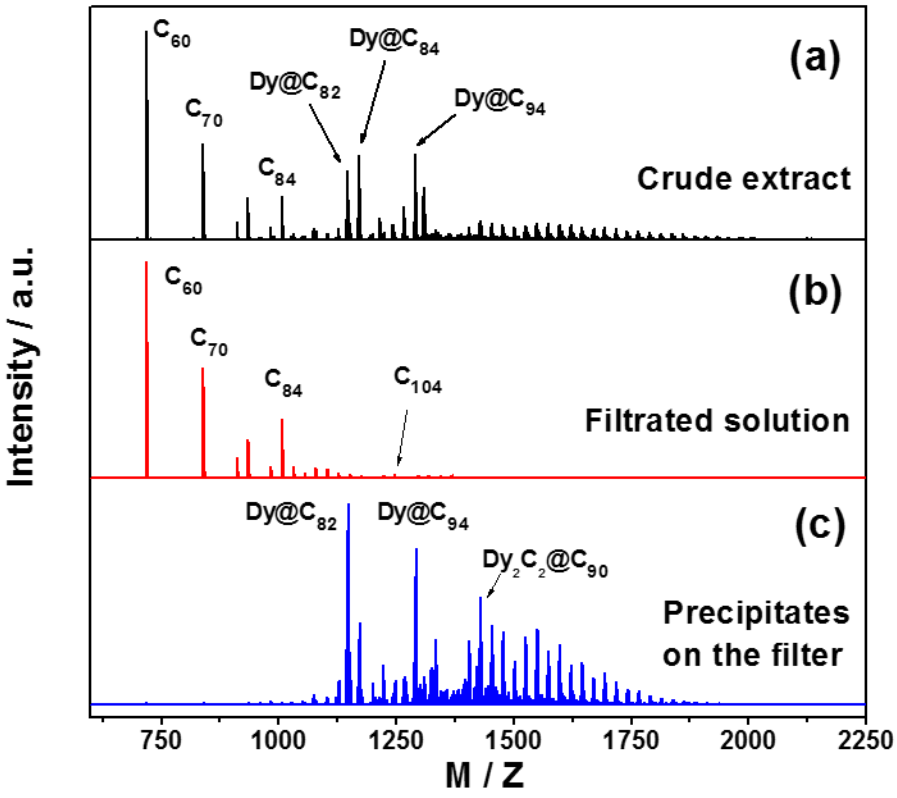

Figure S1. MALDI-TOF of (a) crude extract, (b) filtered solution, and (c) precipitates on the filter for Dy-metallofullerenes.

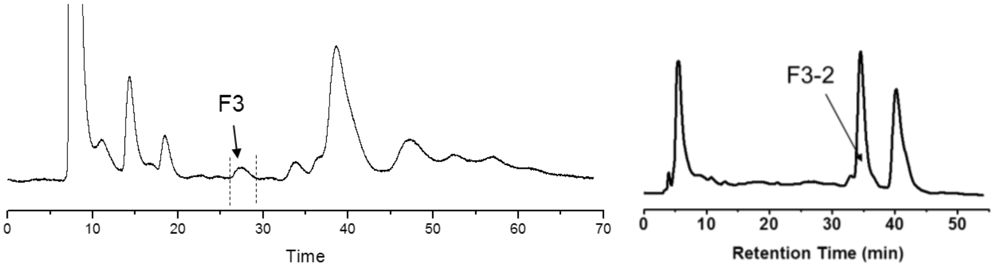

Figure S2. HPLC separation of $Dy_2O@C_s(10528)\text{-}C_{72}$. Left: The first stage HPLC chromatogram of extract on a Buckyprep-M column (Φ = 25 mm × 250 mm, flow rate 10 mL/min). Right: the second stage HPLC chromatogram of fraction F3 on a Buckyprep column (Φ = 10 mm × 250 mm, flow rate 4 mL/min). Fraction F3-2 is $Dy_2O@C_{72}$. Eluent = toluene detecting wavelength = 310 nm.

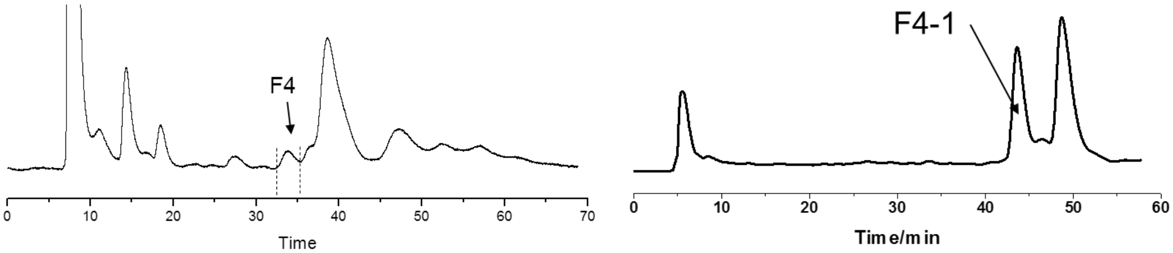

Figure S3. HPLC separation of $Dy_2O@C_2(13333)\text{-}C_{74}$. Left: The first stage HPLC chromatogram of extract on a Buckyprep-M column (Φ = 25 mm × 250 mm, flow rate 10 mL/min). Right: the second stage HPLC chromatogram of fraction F4 on a Buckyprep column (Φ = 10 mm × 250 mm, flow rate 4 mL/min). Fraction F4-1 is $Dy_2O@C_{74}$. Eluent = toluene detecting wavelength = 310 nm.

X-ray analysis

Crystals were grown by layering the benzene solution of nickel octaethylporphyrin (Ni(OEP)) onto the CS_2 solution of the $Dy_2O@C_{2n}$ (2n = 72, 74) isomers. $Dy_2O@C_s(10528)\text{-}C_{72}\cdot Ni(OEP)\cdot 2(C_6H_6)$ and $Dy_2O@C_2(13333)\text{-}C_{74}\cdot Ni(OEP)\cdot C_6H_6\cdot CS_2$ were measured with Bruker APEX II at 120 and 173 K, respectively. The structures were solved using direct methods[1] and refined on F2 using full-matrix least-squares using the SHELXL2015 crystallographic software package.[2] Hydrogen atoms were inserted at calculated positions and constrained with isotropic thermal parameters. The crystal data are presented in Table S1. The data can be obtained free of charge from the Cambridge Crystallographic Data Centre with CCDC Nos. 1974305 and 1974314, respectively.

Table S1. Crystal data

	$Dy_2O@C_s(10528)\text{-}C_{72}\cdot$ $Ni(OEP)\cdot 2(C_6H_6)$	$Dy_2O@C_2(13333)\text{-}C_{74}\cdot$ $Ni(OEP)\cdot(C_6H_6)\cdot CS_2$
**Formula**	C120 H56 Dy2 N4 Ni O	C117 H50 Dy2 N4 Ni O S2
**Formula weight**	1953.39	1975.44
**Color, habit**	Black, block	Black, block
**Crystal system**	triclinic	triclinic
**Space group**	*P-1*	*P 21/c*
***a*, Å**	14.3857(7)	17.914(2)
***b*, Å**	14.7255(6)	16.589(2)
***c*, Å**	19.1301(9)	25.753(3)
***α*, deg**	84.837(2)	90
***β*, deg**	88.064(2)	106.775(4)
***γ*, deg**	61.889(2)	90
**Volume, Å³**	3559.7(3)	7327.4(15)
Z	2	4
***T*, K**	120	173
**Radiation (λ, Å)**	Cu K-α (1.54187)	Cu K-α (1.54187)
**Unique data (R_{int})**	12960	12074
**Parameters**	1198	1174
**Restraints**	963	1166
**Observed data**	11767	8898
**R_1[a]**	0.0953	0.1286
**wR_2[b]**	0.2800	0.3277
**CCDC NO.**	1974305	1974314

[a]For observed data with $I > 2\sigma(I)$, $R_1 = \frac{\sum||F_O|-|F_C||}{\sum|F_O|}$. [b]For all data, $wR_2 = \sqrt{\frac{\sum\left[w(F_O^2-F_C^2)^2\right]}{\sum\left[w(F_O^2)^2\right]}}$.

DFT-based molecular dynamics

DFT-based Born-Oppenheimer molecular dynamics (BOMD) simulations were performed for $Y_2O@C_{72}$ and $Y_2O@C_{74}$ analogs with atomic masses of Dy assigned to Y. Single point energies and forces were calculated at the PBE/TZ2P level using Priroda code.[3] These forces were used to propagate the system in the canonical ensemble (NVT) using Nose-Hoover algorithm as implemented in the Python Atomic Simulation Environment libraries (ASE 3.0).[4] The thermostat temperature was set to 300 K with the characteristic coupling time of 10 fs. The trajectories were propagated for 100 ps using the initial DFT-optimized coordinates as starting points and initial velocities assigned randomly from Maxwell-Boltzmann distribution at 300 K. These trajectories were used to evaluate the spatial distribution of O and Dy atoms at a given unit volume inside the fullerene with discretization of $0.042 \times 0.042 \times 0.042$ Å^3. The probability isosurfaces obtained by this approach for $Y_2O@C_{72}$ and $Y_2O@C_{74}$ are plotted in Figure S4. Metal atoms oscillate only near their optimized positions. Oxygen atoms exhibit higher mobility in the plane perpendicular to the Dy-Dy axis.

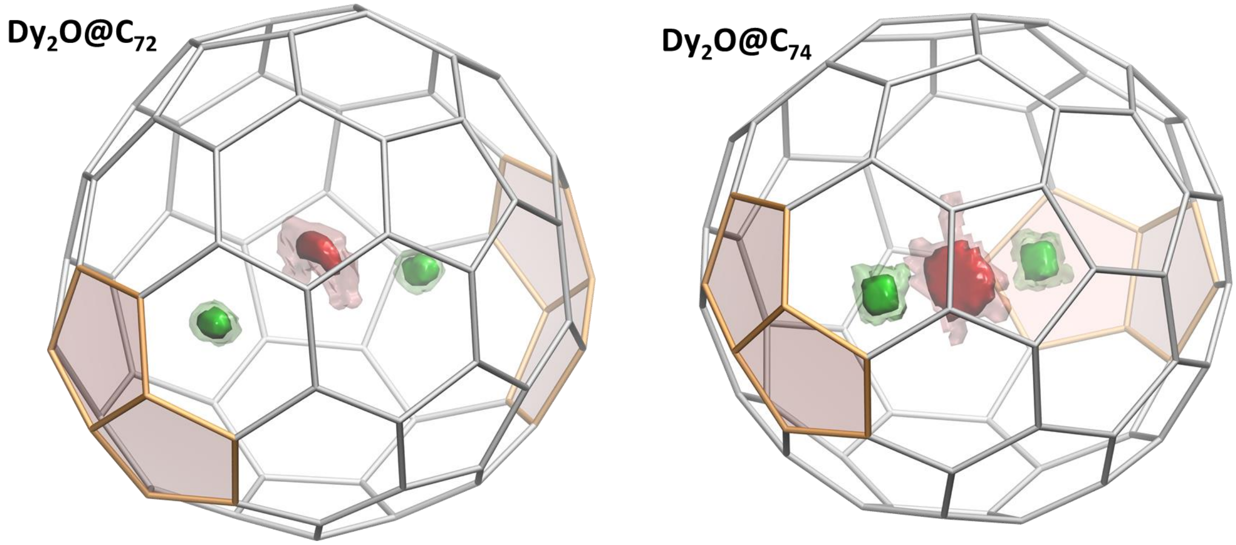

Figure S4. Spatial distribution of the probability density for Dy and O atoms in $Dy_2O@C_{72}$ and $Dy_2O@C_{74}$ as determined from molecular dynamics simulations at T = 300 K. Displacements of carbon atoms are not shown. Two isosurfaces show high probability (solid) and low probability (transparent) volumes.

IR spectra

IR spectra of $Dy_2O@C_{72,74}$ samples drop-casted on KBr substrates were measured at room temperature with Vertex 80 FTIR spectrometer (Bruker) equipped with Hyperion microscope. The spectra were computed using two approaches: in a static approach, molecular coordinates were optimized and then hessian was computed analytically along with derivatives of dipole moment with respect to cartesian coordinates. In molecular dynamics approach, time dependence of the x, y, and z components of the dipole moment obtained in DFT-based molecular dynamics simulations were Fourier-transformed to give corresponding spectra. DFT-computed spectra agree well with the experimental ones. Of particular interest is the identification of the vibrations of the Dy_2O cluster. In the mid-IR range, DFT calculations shows that the Dy−O antisymmetric stretching mode should have relatively high intensity. In the experimental spectra these vibrations can be assigned to medium-intensity absorption bands at 680–700 cm^{-1} (marked by arrows in Fig. S5).

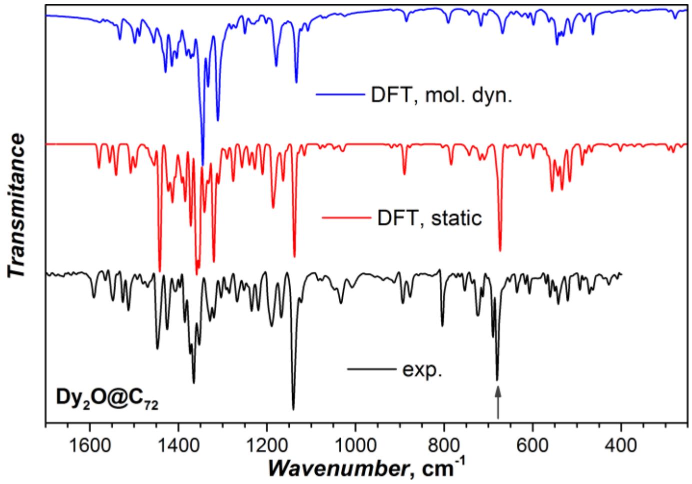

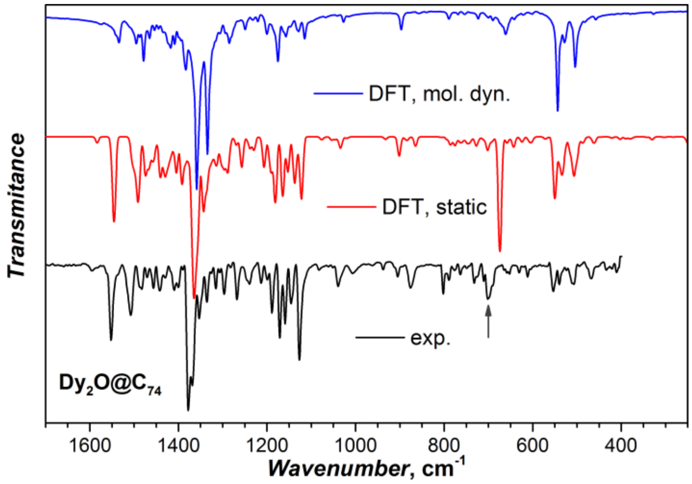

Figure S5. Experimental infrared spectra of $Dy_2O@C_{72}$ and $Dy_2O@C_{72}$ (black) compared to the DFT-computed spectra (from static calculations, red, and from DFT molecular dynamics, blue). Black arrows denote antisymmetric Dy–O stretching mode: the oxygen atom is moving along the line parallel to the Dy···Dy axis so that one Dy–O bond is shortened whereas another one is elongated.

Electrochemical properties

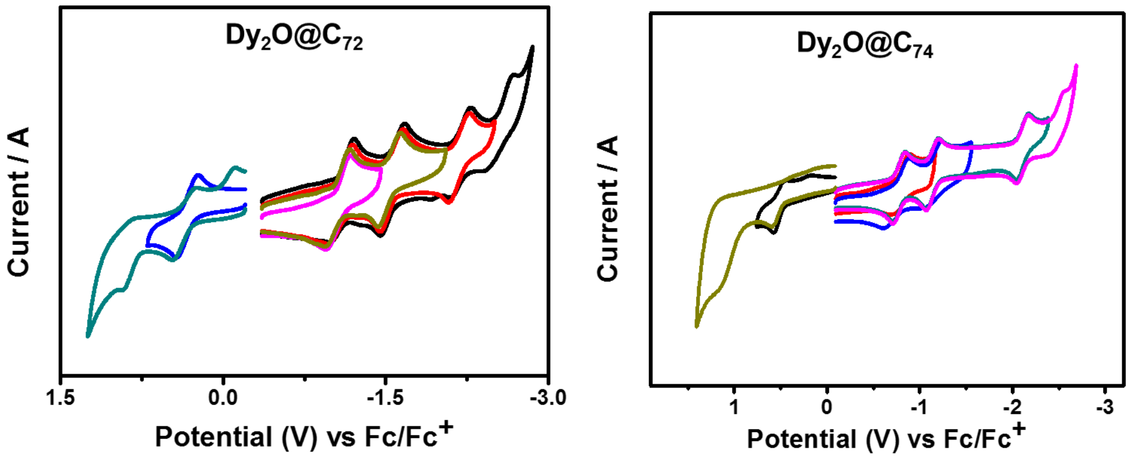

Figure S6. Cyclic voltammetry of $Dy_2O@C_{72}$ (left) and $Dy_2O@C_{74}$ (right) measured in (n-Bu_4)NPF_6/*ortho*-dichlorobenzene solution, potential sweep rate 100 mV/s.

Table S2. Redox potentials of $Dy_2O@C_s$(10528)-C_{72}, $Dy_2O@C_2$(13333)-C_{74} and selected EMFs with the same fullerene cages.

EMF	O-II	O-I	R-I	R-II			R-III	R-IV	gap_{EC}	Ref.
$Dy_2O@C_{72}$	0.87[b]	0.33[a]	−1.09[a]	−1.56[a]			−2.18[a]	−2.55[a]	1.42	t.w.
$Sc_2S@C_{72}$	1.21[b]	0.64[a]	−1.14[a]	−1.53[a]			−2.24[a]		1.78	5
$Sc_2C_2@C_{72}$		0.41[a]	−1.19[a]	−1.54[a]		−1.75[b]	−2.23[a]		1.60	6
$Dy_2O@C_{74}$	1.18[b]	0.52[a]	−0.81[a]	−1.17[a]			−2.16[a]	−2.56[b]	1.33	t.w.
$Ho_2O@C_{74}$		0.47[a]	−0.83[a]	−1.28[b]	−1.60[b]	−1.77[a]	−2.18[a]		1.30	7

[a] Half-wave potential (reversible redox process). [b] Peak potential (irreversible redox process)

Frontier Molecular Orbitals

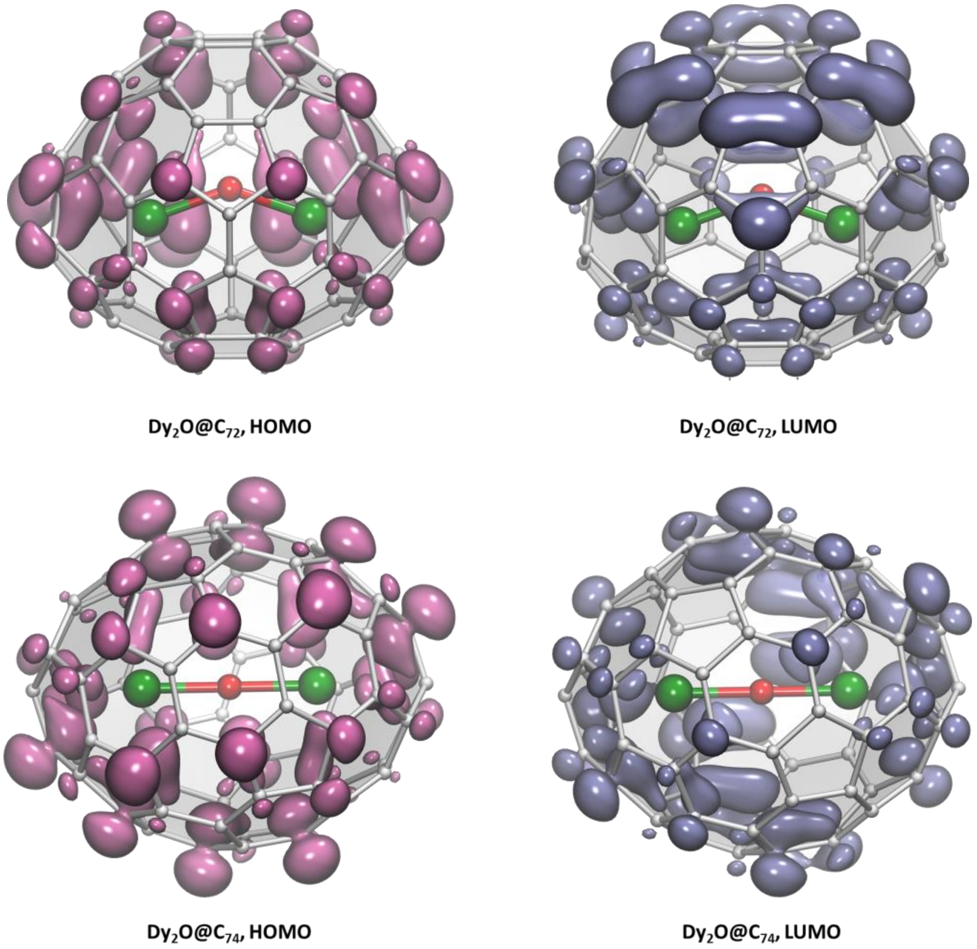

Figure S7. Kohn-Sham molecular orbital densities of the HOMO and LUMO of $Dy_2O@C_{72}$ and $Dy_2O@C_{74}$.

Measurement of magnetic properties

Magnetic properties were measured with MPMP 3 system (Quantum Design). The samples for magnetic measurements were prepared by drop-casting CS_2 solution of metallofullerenes directly onto quartz sample holders. Each sample contained ca 0.1 mg of dried fullerene after evaporation of CS_2. As fullerene molecules are strongly disordered in powders (unless co-crystallization agents are used), magnetic measurements of such powder samples do not require encapsulation in diamagnetic matrix. Besides, quartz holders have negligible diamagnetic signal at helium temperature, which eliminates the need for diamagnetic correction at low temperatures. Since mass of the sample could not be determined precisely, in the fitting of magnetization curves we relied on the shape of the curves rather than on the absolute values.

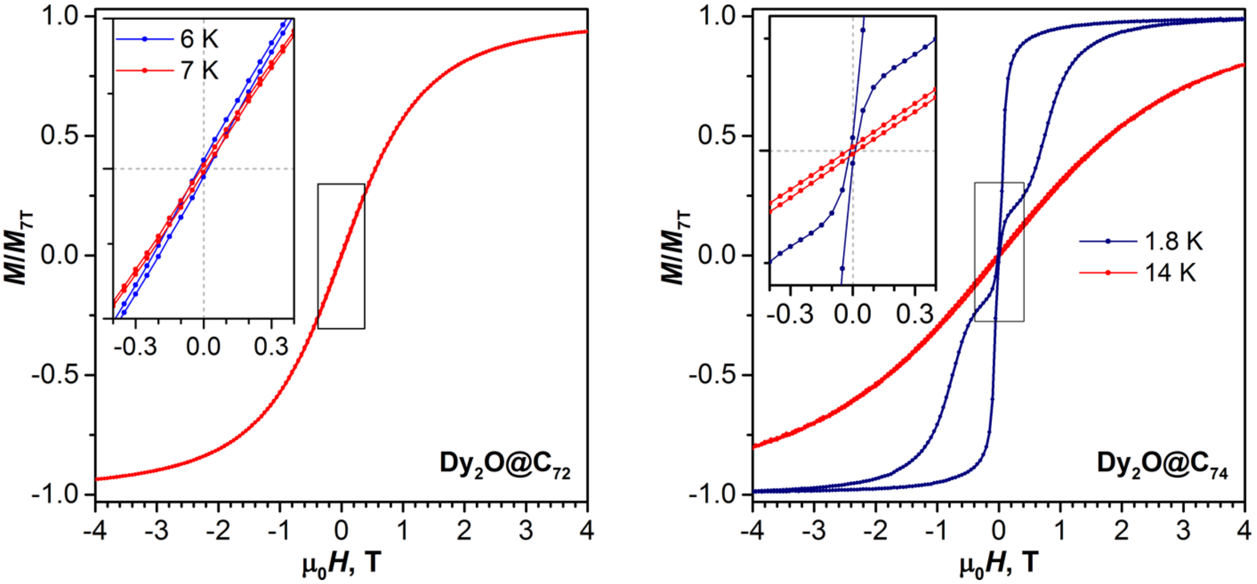

Figure S8. Magnetization curves of $Dy_2O@C_{72}$ (left, 6 and 7 K) and $Dy_2O@C_{74}$ (right, 1.8 K and 14 K), the insets zoom into the region near zero field demonstrating opening of the hysteresis. Magnetic field sweep rate 2.9 mT/s.

ZFC and FC measurements

To study blocking of magnetization in Dy-oxide clusterfullerenes, we performed series of measurements to compare temperature dependence of magnetization for the sample preliminary cooled down to 2 K and then measured during warming up (zero-field cooled, ZFC) and, for the same sample, but the measurement is done during cooling the sample down in the applied field. An example of the measurements sequence is shown in Figure S9, which plots temperature, magnetic field, and magnetization as a function of time. At the moment t_0, magnetic field is zero, and temperature is 30 K. Between t_0 and t_1, the sample is cooled down to 2 K in zero field, magnetization is also essentially zero during this temperature sweep. Then, during the [t_1,t_2] period, the sample is stabilized at T = 2 K for one minute. At the moment t_2, the field is ramped to 0.2 T (it takes ca 2 second to reach this field) and the command to start the temperature sweep is initiated. However, at this moment, magnetometer usually starts additional temperature stabilization, which proceeds between t_2 and t_3, and only then the real temperature sweep is started. Unfortunately, users have no control of the [t_2,t_3] period, and in the measurements shown in Fig. S10 this period varied randomly from 5 to 70 seconds. Since magnetization of the sample is increasing during the [t_2,t_3] period, when the temperature sweep starts at the moment t_3, magnetization is already not zero, but attains some finite value. Depending on the relaxation time of the sample and [t_2,t_3] time, the deviation of the magnetization from zero can be from very small to quite significant. Then, between t_3 and t_4, magnetization is measured during warming the sample up to 30 K (red dots in Fig. S9 and red curves in Fig. S10; this section is referred to as ZFC). Since relaxation of magnetization accelerates with the temperature increase, magnetization (which is smaller than the equilibrium value) first increases till reaches equilibrium value at some temperature, when relaxation become fast enough so that thermodynamic equilibrium is established faster than the temperature is changed. Above this temperature, magnetization decreases with temperature following the equilibrium behavior. After reaching 30 K, the sample is again stabilized between t_4 and t_5, and then the temperature sweep down to 2 K is started and proceeds between t_5 and t_6. Magnetization (referred to as FC, blue dots) during this sweep is increasing. At the beginning of the sweep, this increase follows the thermodynamic behavior, but at some temperature relaxation of magnetization becomes slow. Thus, when temperature reaches 2 K, magnetization is smaller than the equilibrium magnetization for this temperature. If the measurement of magnetization is then continued at the constant temperature ([t_6, t_{fin}] period), gradual increase of magnetization can be observed. Results of such measurements are usually presented as an overlay of ZFC and FC magnetization curves as a function of temperature (e.g., Fig. S10). At higher temperatures, when relaxation of magnetization is fast and thermodynamic equilibrium is restored faster than the temperature is changed, ZFC and FC curves coincide. When relaxation of magnetization becomes slow at lower temperatures and thermodynamic equilibrium is not restored anymore on the temperature sweep timescale, FZC and FC curves bifurcate. Usually, ZFC curve shows a peak near the bifurcation point, and the temperature of the peak is defined the blocking temperature, T_B. Sometimes, ZFC and FC curves also bifurcate above T_B, and then the bifurcation point is defined at T_{irrev}.[8] T_B and T_{irrev} are kinetic parameters and depend on the measurement settings such as magnetic field, temperature sweep rate. Besides, the shape of ZFC curve will also depend strongly on the temperature stabilization time [t_2,t_3]. For the sake of comparison with other fullerene samples, we report the values measured in a field of 0.2 T with the temperature sweep rate of 5 K/min. Figure S10 compare the measurements for $Dy_2O@C_{72}$ in different fields, and also with different lowest temperature (2 K and 3 K). Figure S11 shows the measurements for $Dy_2O@C_{74}$ in different fields.

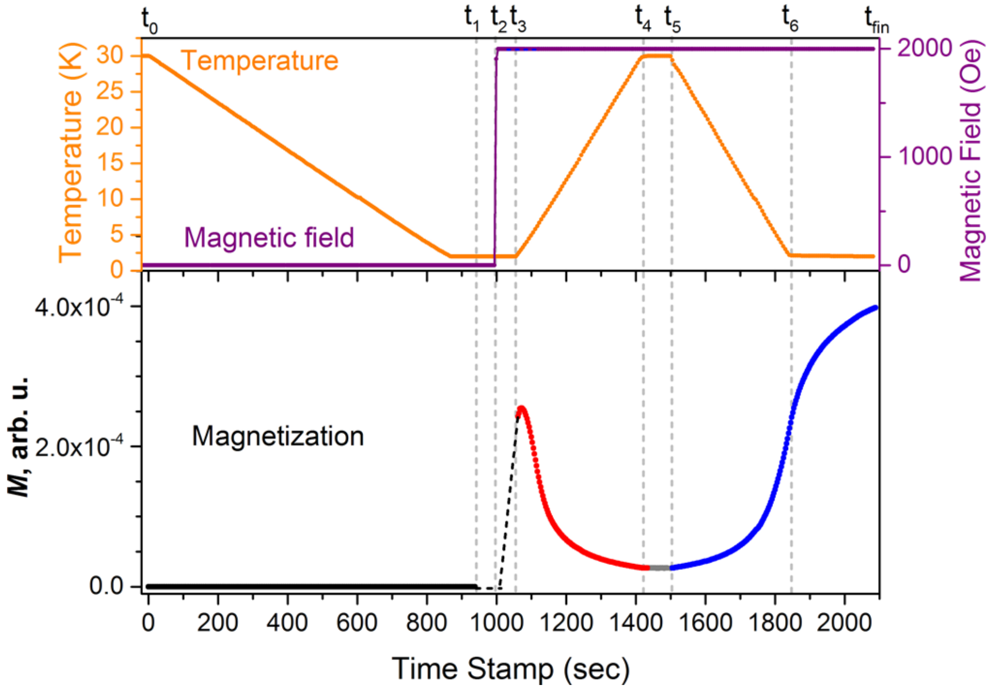

Figure S9. Temperature, magnetic field, and magnetization as a function of time during ZFC/FC measurements of $Dy_2O@C_{72}$ in the field of 0.2 T Colors of magnetization segments correspond to the color of curves in Fig. S10

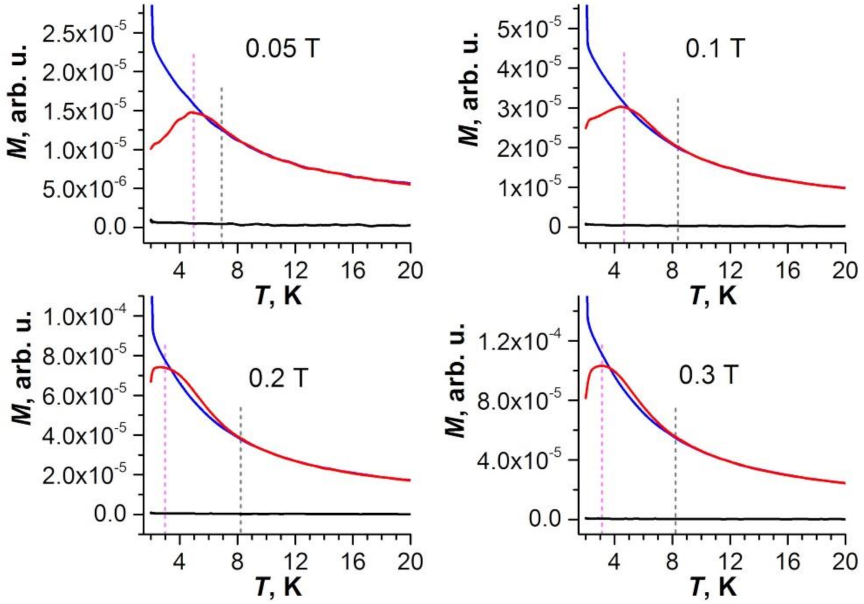

Figure S10a. ZFC and FC measurements for $Dy_2O@C_{72}$ in different magnetic fields, temperature range [2 K, 30 K]. Black curve is magnetization during cooling down in zeo field, red curve – measurement during warming up in the field, blue curve – measurement during cooling down in the field. See Fig. 9 for the measurement sequence. Temperature stabilization time for the measurement in 0.2 T was almost 70 s, which gives unconventional shape of ZFC/FC curves.

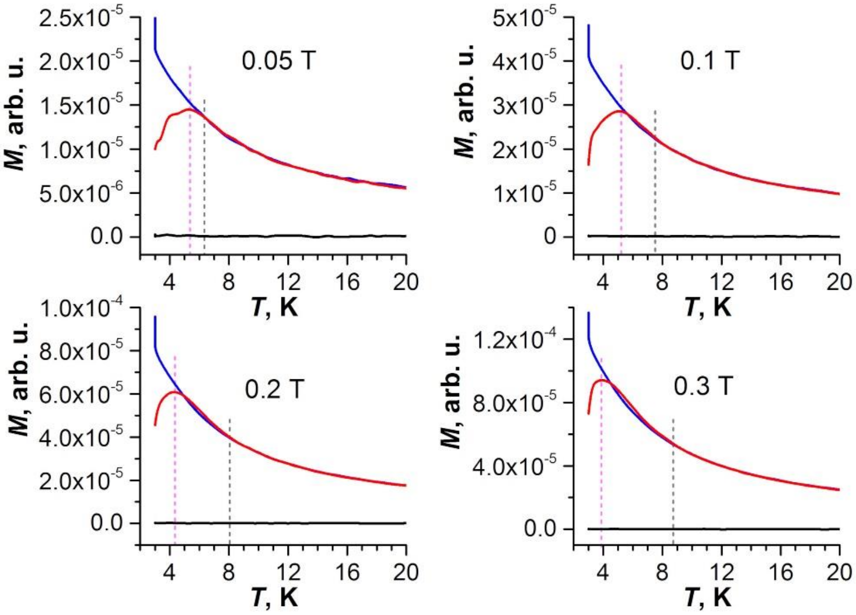

Figure S10b. ZFC and FC measurements for $Dy_2O@C_{72}$ in different magnetic fields. Temperature range [3 K, 30 K]. The only difference from Fig. 10a is that the sample was cooled down to 3 K instead of 2 K in a hope to reduce temperature stabilization time [t_2,t_3] at the start of the ZFC scan.

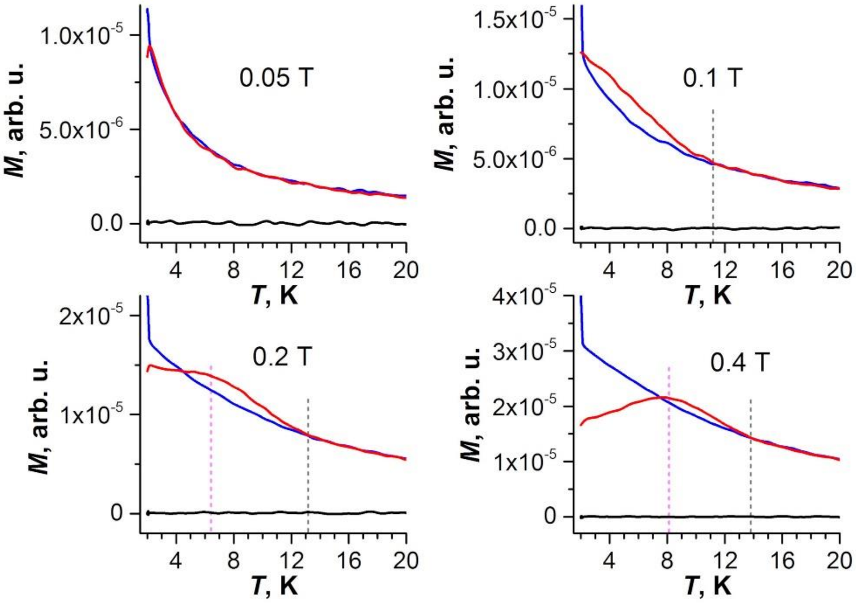

Figure S11. ZFC and FC measurements for $Dy_2O@C_{74}$ in different magnetic fields, temperature range [2 K, 30 K]. Due to the fast relaxation of magnetization of $Dy_2O@C_{74}$ in small fields caused by the QTM (see Fig. 4 in the manuscript of the field dependence of relaxation times), magnetization of $Dy_2O@C_{74}$ always jumps to a relatively high during the field ramp from zero to the measurement field. As a result, ZFC/FC curves strongly depend on the magnetic field used in the measurements. The higher the field- the more conventional the shapes are.

Magnetization relaxation times

Magnetization decay curve was then fitted with stretched exponential function:

$$M(t) = M_{eq} + (M_0 - M_{eq}) \exp\left[-\left(\frac{t}{\tau_M}\right)^{\beta}\right] \quad \text{(S1)}$$

Where M_{eq} and M_0 are the equilibrium and initial magnetizations, respectively, τ_M is a characteristic relaxation time and β is an additional parameter that corresponds to the time-dependent decay rate.

Table S3. Relaxation times of $Dy_2O@C_{72}$ measured in a field of 0 T

***T*, K**	**τ, s**	**±**	**β**	**±**	***M_eq***	**±**	***M_0***	**±**
1.8	522	1	0.812	0.001	-1.5E-6	2.1E-8	2.4E-4	1.1E-7
1.9	443	1	0.789	0.001	2.3E-6	2.6E-8	2.0E-4	1.1E-7
2.0	417	1	0.799	0.001	-1.5E-6	1.8E-8	2.4E-4	1.2E-7
2.1	350	1	0.780	0.001	2.4E-6	4.0E-8	1.9E-4	2.1E-7
2.2	343	1	0.795	0.001	-1.1E-6	2.0E-8	2.3E-4	3.1E-7
2.35	270	1	0.765	0.001	2.0E-6	2.8E-8	1.7E-4	1.6E-7
2.5	255	1	0.793	0.001	-6.2E-7	2.7E-8	2.0E-4	1.6E-7
2.65	195	1	0.755	0.001	2.3E-6	3.3E-8	1.6E-4	1.8E-7
2.8	167	1	0.776	0.001	3.5E-6	4.8E-8	1.5E-4	1.8E-7
3.0	128	1	0.812	0.002	7.1E-6	1.5E-7	1.3E-4	2.5E-7
3.3	112	1	0.744	0.002	-5.3E-7	1.9E-8	1.5E-4	2.6E-7
3.6	75	1	0.799	0.004	4.0E-6	8.4E-8	1.1E-4	2.3E-7
4.0	61	1	0.757	0.004	-1.4E-7	2.8E-8	9.5E-5	2.8E-7

Table S4. Relaxation times of $Dy_2O@C_{72}$ measured in a field of 0.2 T

***T*, K**	**τ, s**	**±**	**β**	**±**	***M_eq***	**±**	***M_0***	**±**
1.8	215	1	0.683	0.003	1.9E-4	1.6E-8	2.8E-4	8.6E-8
1.9	182	1	0.776	0.003	1.7E-4	8.7E-8	2.3E-4	9.8E-8
2.0	171	1	0.684	0.003	1.8E-4	1.5E-8	2.7E-4	1.2E-7
2.1	153	1	0.742	0.002	1.5E-4	6.8E-8	2.2E-4	1.1E-7
2.2	146	1	0.698	0.003	1.6E-4	1.8E-8	2.5E-4	2.5E-7
2.35	127	1	0.741	0.002	1.4E-4	5.8E-8	2.1E-4	1.5E-7
2.5	116	1	0.700	0.003	1.5E-4	1.7E-8	2.3E-4	1.4E-7
2.65	94	1	0.786	0.003	1.3E-4	1.1E-7	1.9E-4	1.3E-7
2.8	85	1	0.736	0.003	1.2E-4	1.1E-7	1.8E-4	1.5E-7
3.0	75	1	0.751	0.004	1.1E-4	1.0E-7	1.7E-4	1.7E-7
3.3	65	1	0.731	0.005	1.1E-4	1.9E-8	1.8E-4	3.1E-7
3.6	50	1	0.713	0.003	9.5E-5	4.6E-8	1.5E-4	2.0E-7
4.0	37	1	0.658	0.004	9.1E-5	1.2E-8	1.4E-4	2.1E-7

Table S5. Relaxation times of $Dy_2O@C_{72}$ measured in a temperature of 1.8 K

μ_0H, T	τ, s	±	β	±	M_{eq}	±	M_0	±
0	523	1	0.812	0.001	-1.5E-6	2.1E-8	2.4E-4	1.1E-7
0.1	405	1	0.807	0.002	1.1E-4	2.6E-7	2.3E-4	1.3E-7
0.15	290	1	0.760	0.002	1.4E-4	1.0E-7	2.3E-4	1.3E-7
0.2	215	1	0.683	0.003	1.9E-4	1.6E-8	2.8E-4	8.6E-8
0.25	161	1	0.716	0.003	1.6E-4	1.8E-8	2.4E-4	1.4E-7
0.3	113	1	0.809	0.004	2.2E-4	8.2E-8	2.5E-4	7.8E-8
0.4	95	1	0.825	0.010	2.55E-4	8.07E-8	2.62E-4	6.68E-8
0.5	86	1	0.832	0.012	2.69E-4	5.90E-8	2.73E-4	8.10E-8

Table S6. Relaxation times of $Dy_2O@C_{74}$ measured in a field of 0.2 T

T, K	τ, s	±	β	±	M_{eq}	±	M_0	±
1.8	1606	8	0.545	0.002	5.7E-5	8.6E-8	1.0E-4	5.2E-8
1.9	1513	6	0.549	0.001	5.2E-5	7.0E-8	1.6E-4	7.5E-8
2	1332	4	0.568	0.001	5.3E-5	5.4E-8	9.9E-5	4.7E-8
2.1	1254	3	0.565	0.001	4.8E-5	4.2E-8	9.8E-5	6.8E-8
2.2	1102	2	0.583	0.001	4.9E-5	3.4E-8	9.8E-5	5.8E-8
2.35	981	2	0.601	0.001	4.5E-5	3.4E-8	9.4E-5	4.9E-8
2.5	805	1	0.614	0.001	4.5E-5	2.3E-8	9.5E-5	5.6E-8
2.65	718	1	0.623	0.001	4.1E-5	3.7E-8	9.2E-5	8.5E-8
2.8	598	1	0.627	0.001	4.1E-5	1.5E-8	9.2E-5	5.7E-8
3.1	455	1	0.663	0.001	3.7E-5	2.9E-8	8.7E-5	5.5E-8
3.3	334	1	0.669	0.001	5.7E-5	2.9E-8	1.3E-4	8.7E-8
3.65	268	1	0.718	0.001	3.3E-5	3.2E-8	7.9E-5	6.5E-8
4	187	1	0.729	0.002	4.9E-5	3.1E-8	1.1E-4	9.0E-8
4.5	140	1	0.746	0.001	2.7E-5	1.8E-8	6.9E-5	8.2E-8
5	97	1	0.758	0.002	4.1E-5	1.8E-8	9.3E-5	1.2E-7
5.5	78	1	0.791	0.003	2.3E-5	2.9E-8	5.5E-5	9.4E-8
6	61	1	0.778	0.003	2.1E-5	1.9E-8	4.9E-5	9.4E-8

Table S7. Relaxation times of $Dy_2O@C_{74}$ measured in a field of 0.8 T

T, K	*τ*, s	±	β	±	*M_{eq}*	±	*M₀*	±
1.8	753	4	0.568	0.003	1.8E-4	8.9E-9	1.9E-4	1.6E-7
1.9	719	8	0.578	0.005	1.1E-4	3.2E-8	1.1E-4	2.1E-7
2	606	3	0.567	0.002	1.8E-4	9.3E-9	1.9E-4	1.0E-7
2.1	604	5	0.572	0.004	1.0E-4	2.6E-8	1.1E-4	9.2E-8
2.2	525	3	0.588	0.002	1.7E-4	9.4E-9	1.9E-4	1.4E-7
2.35	523	4	0.600	0.005	1.0E-4	2.4E-8	1.1E-4	8.3E-8
2.5	401	2	0.609	0.003	1.7E-4	1.3E-8	1.8E-4	1.5E-7
2.65	395	2	0.637	0.004	9.9E-5	2.5E-8	1.1E-4	1.7E-7
2.8	305	1	0.620	0.002	1.6E-4	1.1E-8	1.8E-4	1.4E-7
3.1	261	1	0.660	0.004	9.4E-5	3.0E-8	1.1E-4	1.1E-7
3.3	223	1	0.656	0.002	1.5E-4	1.1E-8	1.7E-4	1.1E-7
3.65	169	1	0.711	0.004	8.8E-5	2.9E-8	1.0E-4	1.5E-7
4	140	1	0.701	0.002	1.4E-4	1.1E-8	1.6E-4	1.5E-7
4.5	110	1	0.704	0.004	7.8E-5	4.6E-8	9.1E-5	1.0E-7
5	82	1	0.743	0.002	1.2E-4	6.9E-9	1.4E-4	1.5E-7
5.5	66	1	0.748	0.004	6.9E-5	1.7E-8	8.0E-5	1.1E-7
6	52	1	0.673	0.009	6.5E-5	3.4E-8	7.5E-5	1.2E-7

Table S8. Relaxation times of $Dy_2O@C_{74}$ measured in a temperature of 2.5 T

μ₀H, T	*τ*, s	±	β	±	*M_{eq}*	±	*M₀*	±
0.05	152	1	0.554	0.004	1.4E-5	5.6E-8	3.3E-5	1.5E-7
0.1	254	1	0.578	0.002	2.6E-5	5.9E-8	6.6E-5	8.0E-8
0.125	440	1	0.578	0.002	3.0E-5	4.5E-8	7.7E-5	6.6E-8
0.15	565	1	0.601	0.001	3.6E-5	5.4E-8	8.4E-5	1.7E-7
0.175	685	1	0.606	0.001	4.0E-5	6.2E-8	9.0E-5	6.8E-8
0.2	747	1	0.681	0.001	6.7E-5	2.5E-8	9.1E-5	4.7E-8
0.25	781	1	0.612	0.001	4.3E-5	1.6E-8	9.5E-5	3.7E-8
0.3	773	1	0.614	0.001	4.3E-5	2.5E-8	9.8E-5	3.4E-8
0.35	753	1	0.694	0.001	6.7E-5	2.2E-8	1.0E-4	1.3E-7
0.375	712	1	0.693	0.001	7.0E-5	1.8E-8	1.0E-4	4.2E-8
0.4	621	1	0.694	0.001	7.4E-5	2.6E-8	1.0E-4	6.2E-8
0.45	568	1	0.699	0.001	8.0E-5	2.4E-8	1.1E-4	6.1E-8
0.5	510	1	0.696	0.002	8.5E-5	2.1E-8	1.1E-4	8.0E-8
0.6	438	1	0.654	0.002	9.2E-5	2.0E-8	1.1E-4	1.0E-7
0.7	414	2	0.643	0.004	9.9E-5	3.0E-8	1.1E-4	1.1E-7
0.8	398	2	0.602	0.003	1.0E-4	1.7E-8	1.1E-4	9.6E-8
0.9	371	2	0.628	0.004	1.1E-4	1.7E-8	1.2E-4	1.3E-7
1	369	3	0.633	0.008	1.1E-4	2.5E-8	1.2E-4	1.3E-7
1.1	342	3	0.578	0.009	1.17E-4	1.87E-8	1.18E-4	1.28E-7
1.2	338	3	0.631	0.007	1.16E-4	1.69E-8	1.19E-4	1.48E-7

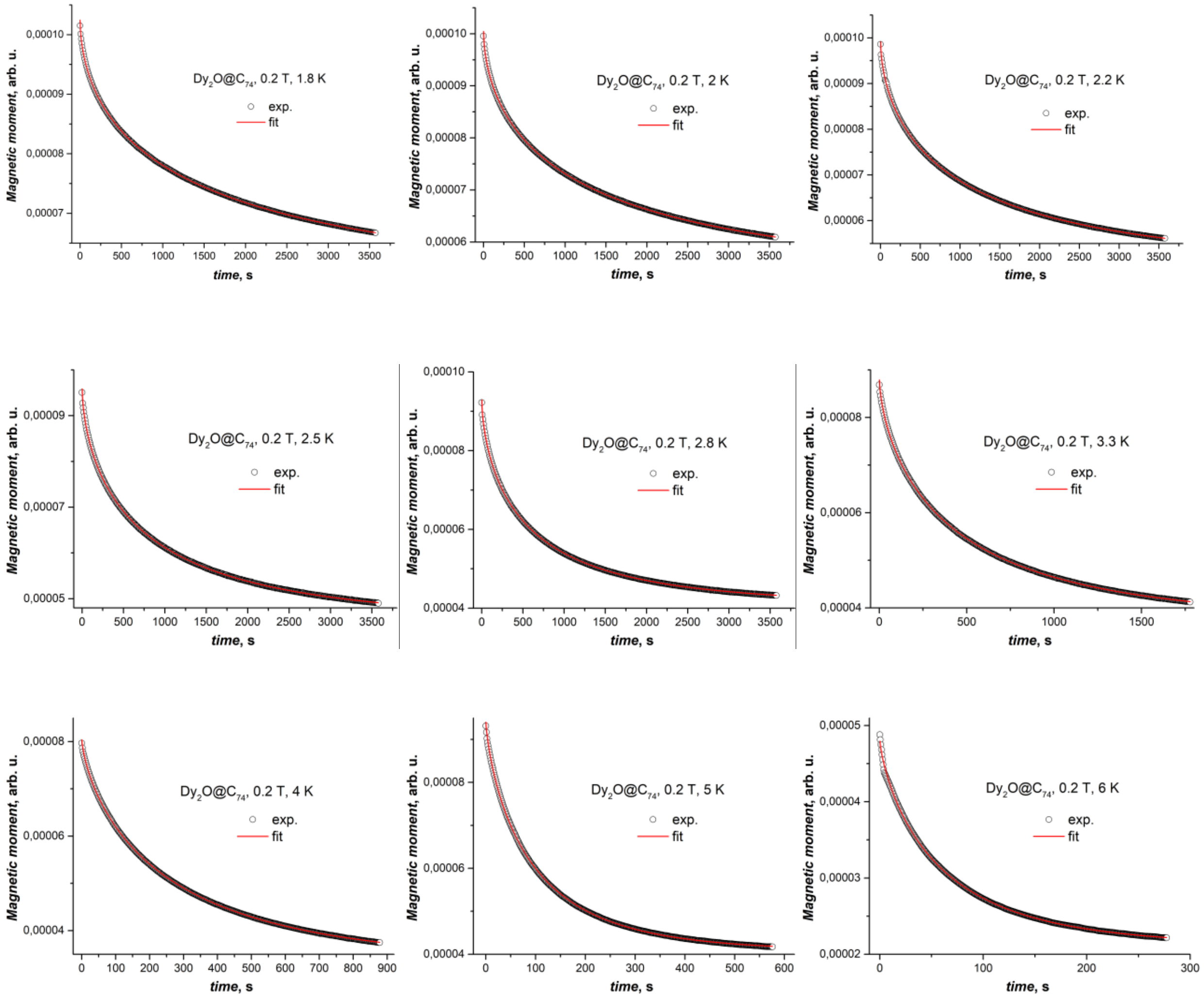

Figure S12. Selected magnetization decay curves of $Dy_2O@C_{74}$ measured at different temperatures in a field of 0.2 T. The complete set of determined values is listed in Table S6.

Temperature dependence of relaxation times

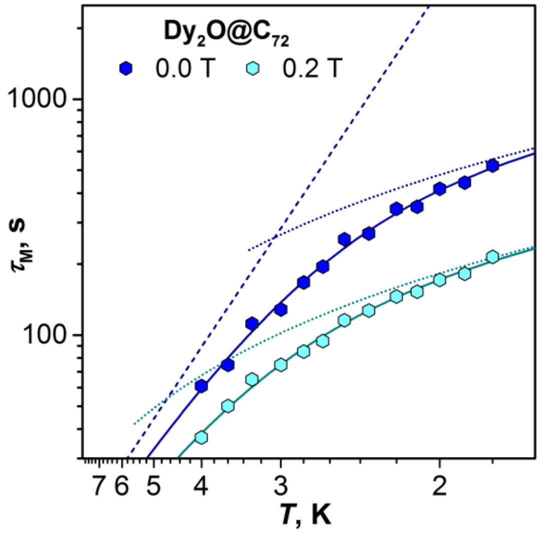

Figure S13a. Magnetization relaxation times of $Dy_2O@C_{72}$ measured in a field of 0 T and 0.2 T (dots). Solid lines are fits with the model:

$$C_{\mathrm{d},H}T^{n_\mathrm{d}} + \tau_0^{-1}\exp(-U^{\mathrm{eff}}/T)$$

Dashed line is $\tau_0^{-1}\exp(-U^{\mathrm{eff}}/T)$ contribution, dotted lines are $C_{\mathrm{d},H}T^{n_\mathrm{d}}$ functions for different fields. Fitted parameters are:

n_d=1.44±0.13

$C_{\mathrm{d,0T}}$ = (7.7±0.6)·10^{-4} $s^{-1}K^{-1.44}$

$C_{\mathrm{d,0.2T}}$ = (2.02±0.19)·10^{-3} $s^{-1}K^{-1.44}$

τ_0=2.7±0.7 s

U^{eff}= 14±1 K

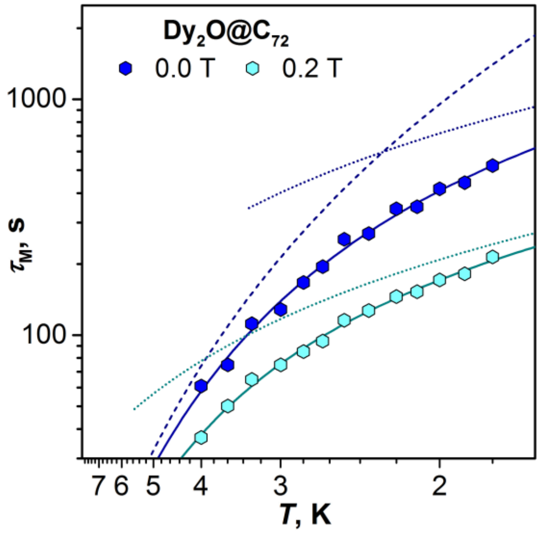

Figure S13b. Magnetization relaxation times of $Dy_2O@C_{72}$ measured in a field of 0 T and 0.2 T (dots). Solid lines are fits with the model:

$$\tau_\mathrm{M}^{-1}(T) = C_{\mathrm{d},H}T^{n_\mathrm{d}} + C_\mathrm{R}T^{n_R}$$

Dashed line is $C_\mathrm{R}T^{n_R}$ contribution, dotted lines are $C_{\mathrm{d},H}T^{n_\mathrm{d}}$ functions for different fields. Fitted parameters are:

n_d=1.43±0.13

$C_{\mathrm{d,0T}}$ = (5.2±0.1)·10^{-4} $s^{-1}K^{-1.43}$

$C_{\mathrm{d,0.2T}}$ = (1.78±0.18)·10^{-3} $s^{-1}K^{-1.43}$

n_R=3.69±0.36

C_R = (8.2±4.5)·10^{-5} $s^{-1}K^{-3.7}$

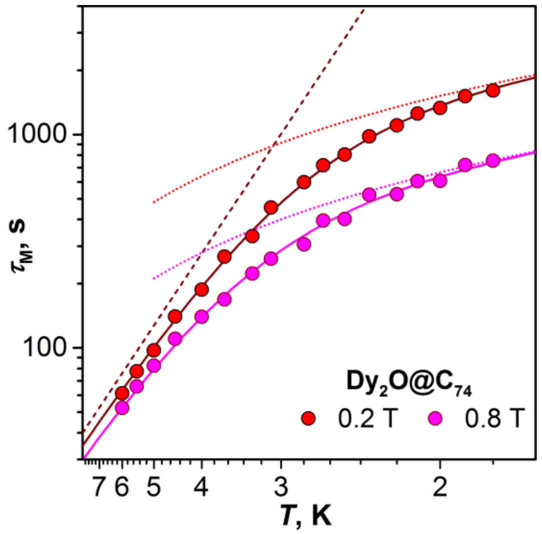

Figure S14a. Magnetization relaxation times of $Dy_2O@C_{74}$ measured in a field of 0.2 T and 0.8 T (dots). Solid lines are fits with the model:

$$C_{\mathrm{d},H}T^{n_\mathrm{d}} + \tau_0^{-1}\exp(-U^{\mathrm{eff}}/T)$$

Dashed line is $\tau_0^{-1}\exp(-U^{\mathrm{eff}}/T)$ contribution, dotted lines are $C_{\mathrm{d},H}T^{n_\mathrm{d}}$ functions for different fields. Fitted parameters are:

n_d = 1.25±0.10
$C_{\mathrm{d,0.2T}}$ = (2.77±0.17)·10^{-4} s^{-1}K$^{-1.25}$
$C_{\mathrm{d,0.8T}}$ = (6.32±0.49)·10^{-4} s^{-1}K$^{-1.25}$
τ_0 = 5.75±0.45 s
U^{eff} = 15.5±0.5 K

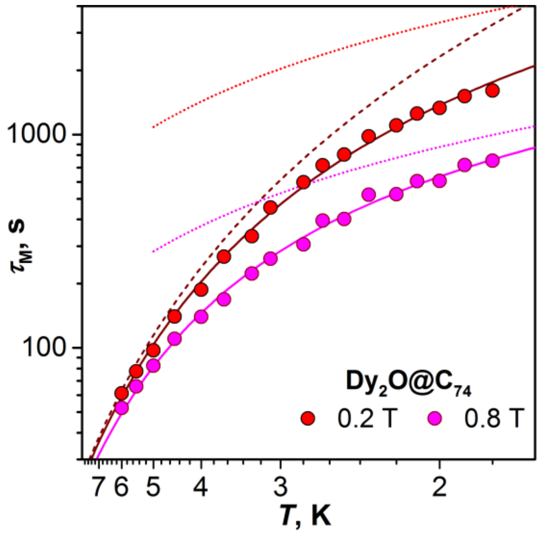

Figure S14b. Magnetization relaxation times of $Dy_2O@C_{74}$ measured in a field of 0.2 T and 0.8 T (dots). Solid lines are fits with the model:

$$\tau_\mathrm{M}^{-1}(T) = C_{\mathrm{d},H}T^{n_\mathrm{d}} + C_\mathrm{R}T^{n_R}$$

Dashed line is $C_\mathrm{R}T^{n_R}$ contribution, dotted lines are $C_{\mathrm{d},H}T^{n_\mathrm{d}}$ functions for different fields. Fitted parameters are:

n_d = 1.23±0.14
$C_{\mathrm{d,0.2T}}$ = (1.27±0.29)·10^{-4} s^{-1}K$^{-1.23}$
$C_{\mathrm{d,0.8T}}$ = (4.88±0.51)·10^{-4} s^{-1}K$^{-1.23}$
n_R = 3.28±0.14
C_R = (4.4±1.1)·10^{-5} s^{-1}K$^{-3.28}$

Ab initio computed ligand-field splitting

Table S9. Energies and composition in $|m_J\rangle$ basis of the ligand-field states of Dy^{3+} ions in $Dy_2O@C_{72}$ and $Dy_2O@C_{74}$.

KD	E, cm^{-1}	**$Dy_2O@C_{72}$,** Composition, %[a]	E, cm^{-1}	**$Dy_2O@C_{74}$,** Composition, %[a]
1	0	$99.4\|15/2\rangle$	0	$99.5\|15/2\rangle$
2	340	$98.0\|13/2\rangle$	336	$98.9\|13/2\rangle$
3	717	$94.4\|11/2\rangle + 3.8\|9/2\rangle + 1.5\|13/2\rangle$	716	$97.4\|11/2\rangle + 1.7\|9/2\rangle$
4	1029	$88.4\|9/2\rangle + 4.8\|7/2\rangle + 3.4\|11/2\rangle$	1033	$94.7\|9/2\rangle + 2.0\|7/2\rangle$
5	1180	$49.0\|7/2\rangle + 11.8\|-1/2\rangle + 16.5\|-7/2\rangle$	1200	$62.5\|7/2\rangle + 16.2\|-7/2\rangle + 14.4\|1/2\rangle$
6	1239	$32.9\|5/2\rangle + 24.3\|-1/2\rangle + 21.5\|-5/2\rangle$	1261	$54.9\|5/2\rangle + 5.0\|-7/2\rangle + 27.0\|-1/2\rangle$
7	1284	$51.2\|3/2\rangle + 17.6\|-3/2\rangle + 14.3\|1/2\rangle$	1293	$77.5\|3/2\rangle + 6.5\|5/2\rangle + 4.9\|-3/2\rangle$
8	1337	$32.0\|5/2\rangle + 21.7\|-1/2\rangle + 12.1\|1/2\rangle$	1329	$45.6\|1/2\rangle + 28.5\|-5/2\rangle + 9.3\|-3/2\rangle$

[a] only 3 largest components are listed

Fitting of magnetization curves

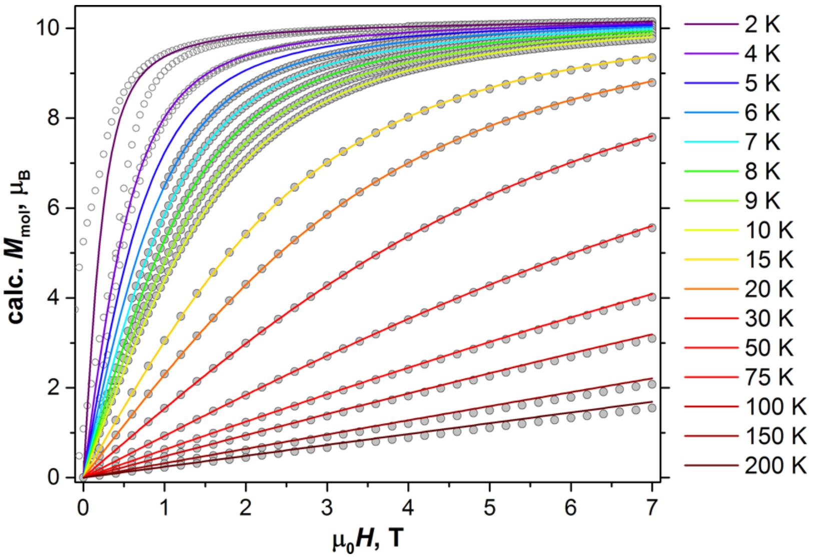

Figure S15. Experimental magnetization values of $Dy_2O@C_{72}$ (gray dots) measured at different temperatures compared to the results of the fitting with Hamiltonian in Eq. (3) (colored lines). Experimental values for the fitting were taken only from the regions where hysteresis is closed. For comparison, open dots show experimental magnetization curves with open hysteresis measured at 2 and 4 K. The optimal $j_{1,2}$ value obtained from the fit is 0.0091 cm^{-1}.

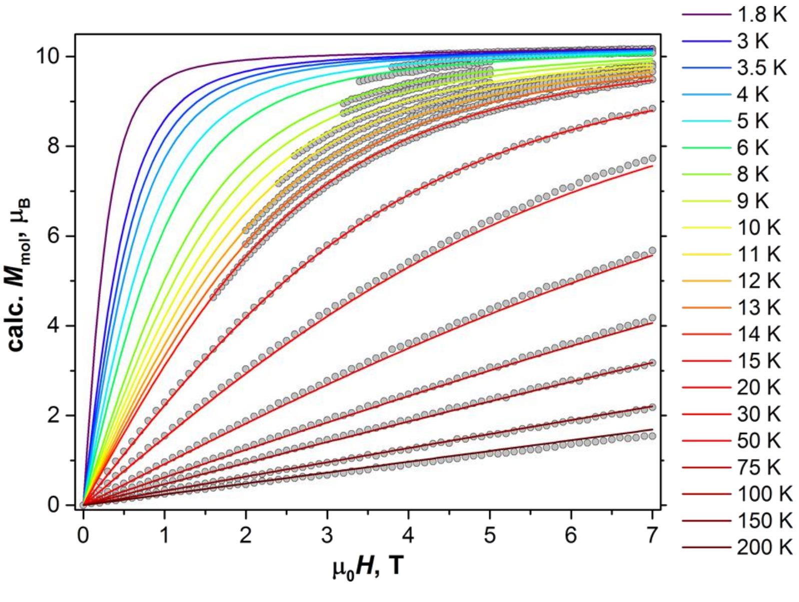

Figure S16. Experimental magnetization values of $Dy_2O@C_{74}$ (gray dots) measured at different temperatures compared to the results of the fitting with Hamiltonian in Eq. (3) (colored lines). Experimental values for the fitting were taken only from the regions where hysteresis is closed (note that for some temperature the measurements were performed only up to 5 T). The optimal $j_{1,2}$ value obtained from the fit is 0.00016 cm^{-1} (if only the T≤14 K is used) and 0.00082 cm^{-1} (if the whole temperature range is used, the curves are shown in the figure). Variation of j_{12} from −0.002 to +0.002 cm^{-1} does not change the curves noticeably.

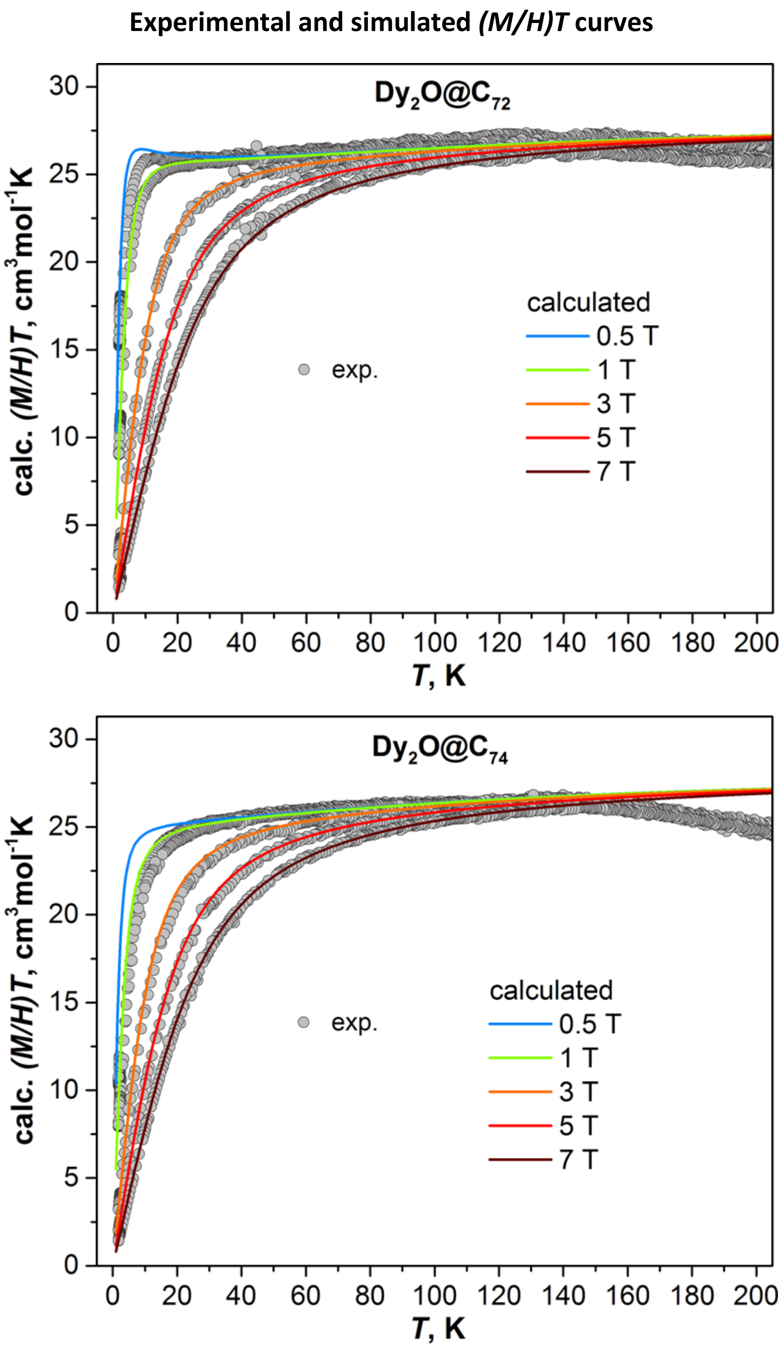

Figure S17. Experimental (dots) and simulated (lines) *(M/H)T* curves. Note that below T_{irrev}, *(M/H)T* curves do not represent thermodynamic equilibrium and hence cannot be well compared to the simulated data. Simulations were performed using coupling constants from the fits of magnetization curves (Fig. S15-S16).

DFT-optimized Cartesian coordinates

$Dy_2O@C_s(10528)-C_{72}$

C	4.201474630	1.314893155	-0.060071591
C	4.135936693	0.515826447	1.137784894
C	4.075449220	0.418356402	-1.189147535
C	3.962868871	-0.864879550	0.782546433
C	3.945697044	-0.945400881	-0.681144128
C	3.306540319	2.449588394	0.109533893
C	3.228537895	1.136014604	2.067817938
C	3.200968976	0.805921872	-2.246639853
C	3.119216944	-1.935786949	-1.348620386
C	3.104080279	-1.708758629	1.578034142
C	2.754650242	2.371234482	1.461773720
C	2.433942170	2.042021064	-2.172213067
C	2.418608988	2.834567094	-0.964648222
C	2.605057673	-2.980232652	-0.531894839
C	2.592076535	-2.863285593	0.918310950
C	2.405375790	-0.168152189	-2.952035033
C	2.354987264	0.340913739	2.887255464
C	2.360860981	-1.548547863	-2.551821223
C	2.337382482	-1.119937909	2.699061496
C	1.457473051	2.905766080	1.791846025
C	1.226922750	1.850276105	-2.947431692
C	1.222959896	0.499851126	-3.435551718
C	1.427329454	-3.727572729	-0.916245634
C	1.418943036	-3.544311118	1.398423931
C	1.166275147	3.456328128	-0.617666667
C	1.173402647	0.997057079	3.359420317
C	1.176972258	-2.271539264	-2.879769337
C	1.181207581	-1.826833441	3.140146879
C	0.701496084	-4.093886857	0.270042564
C	0.719505371	3.551689458	0.748847705
C	0.726391973	2.260168203	2.828366379
C	0.724410165	-3.368635273	-2.070819643
C	0.727928947	-3.025791950	2.490325247
C	-0.000244598	-1.603420939	-3.387296607
C	0.000123265	-1.134034665	3.597572460
C	-0.000257903	-0.205931649	-3.627718349
C	0.000122831	0.247889585	3.699954282
C	-0.000064840	2.500336099	-2.608671983
C	0.000029902	3.344703568	-1.453019491
C	-0.727824724	-3.025769361	2.490397786
C	-0.724939650	-3.368501518	-2.070728526
C	-0.726090028	2.260182888	2.828502577

C	-0.719186129	3.551660558	0.748945994
C	-0.701607956	-4.093863358	0.270108608
C	-1.181042746	-1.826791138	3.140302923
C	-1.177465688	-2.271410141	-2.879667016
C	-1.173175015	0.997067892	3.359630376
C	-1.166134390	3.456427326	-0.617514125
C	-1.418906936	-3.544132604	1.398533880
C	-1.427565097	-3.727523323	-0.915995119
C	-1.223368579	0.499927756	-3.435301447
C	-1.227147787	1.850322422	-2.947136254
C	-1.457134937	2.905811917	1.791998778
C	-2.337265438	-1.119868134	2.699319683
C	-2.361297841	-1.548442972	-2.551656016
C	-2.354815380	0.340967682	2.887550972
C	-2.405757748	-0.167998918	-2.951681661
C	-2.592119980	-2.863160477	0.918517167
C	-2.605216246	-2.980225661	-0.531725344
C	-2.418604338	2.834926676	-0.964402448
C	-2.434067198	2.042208932	-2.171809638
C	-2.754373787	2.371325620	1.461993220
C	-3.104033230	-1.708675812	1.578328467
C	-3.119466859	-1.935784884	-1.348451892
C	-3.201169978	0.806116218	-2.246202490
C	-3.228283225	1.136070033	2.068034597
C	-3.306403882	2.449680465	0.109800381
C	-3.945746695	-0.945439105	-0.680867042
C	-3.962848074	-0.864816557	0.782881627
C	-4.075581904	0.418393088	-1.188782936
C	-4.135729021	0.515884954	1.138038136
C	-4.201386084	1.314945668	-0.059773620
Dy	-1.892022402	0.359401047	-0.015973205
Dy	1.892103482	0.359250204	-0.015879046
O	0.000006706	-0.362594673	-0.000526025

$Dy_2O@C_2(13333)-C_{74}$

C	3.226247926	-2.975273960	0.122298705
C	2.402607954	-3.240077898	-0.995712293
C	1.040871375	-3.699356973	-0.827825929
C	0.293915721	-3.270150653	-1.979200373
C	-1.112406781	-3.042096258	-1.912382552
C	-1.663479361	-2.032690859	-2.759313085
C	-2.823967151	-1.286037727	-2.362445213
C	-2.680192236	0.058957148	-2.869663220
C	-3.256951189	1.191222001	-2.195465239
C	-2.459858519	2.401176604	-2.182100230
C	-2.402607954	3.240077898	-0.995712293
C	-1.040871375	3.699356973	-0.827825929
C	-0.428287678	3.777994352	0.465502361
C	0.990436871	3.647773564	0.500080788
C	1.654628869	3.048354198	1.625962182
C	2.819151314	2.338541264	1.170941818
C	3.235769717	1.123297938	1.796398769
C	4.036035031	0.244999343	0.978903837
C	4.104349696	-1.155898283	1.263660635
C	4.170939711	-1.887623406	0.028845743
C	2.578372522	-2.916550592	1.432611367
C	3.119378923	-1.754179772	2.122858719
C	2.290908947	-0.927022924	2.956086219
C	2.405396387	0.541283659	2.856915677
C	1.296776036	1.310352429	3.315148796
C	0.923459997	2.550933405	2.697248806
C	-0.520569799	2.685224834	2.703005925
C	-1.204888751	3.316064376	1.624624022
C	-2.578372522	2.916550592	1.432611367
C	-3.226247926	2.975273960	0.122298705
C	-4.170939711	1.887623406	0.028845743
C	-4.124065185	0.934023332	-1.064830007
C	-4.104620002	-0.403617598	-0.463920598
C	-3.449196218	-1.518626062	-1.098414538
C	-2.910733971	-2.532105929	-0.254945529
C	-1.753318972	-3.300689098	-0.666800316
C	-0.990436871	-3.647773564	0.500080788
C	0.428287678	-3.777994352	0.465502361
C	1.204888751	-3.316064376	1.624624022
C	0.520569799	-2.685224834	2.703005925
C	1.050905983	-1.510564214	3.347876626
C	-0.070450483	-0.688282983	3.738681547
C	0.070450483	0.688282983	3.738681547
C	-1.050905983	1.510564214	3.347876626
C	-2.290908947	0.927022924	2.956086219
C	-3.119378923	1.754179772	2.122858719
C	-4.104349696	1.155898283	1.263660635

C	-4.036035031	-0.244999343	0.978903837
C	-3.235769717	-1.123297938	1.796398769
C	-2.819151314	-2.338541264	1.170941818
C	-1.654628869	-3.048354198	1.625962182
C	-0.923459997	-2.550933405	2.697248806
C	-1.296776036	-1.310352429	3.315148796
C	-2.405396387	-0.541283659	2.856915677
C	2.459858519	-2.401176604	-2.182100230
C	1.150509429	-2.443901338	-2.795128659
C	0.614897270	-1.321131576	-3.489907929
C	-0.807027337	-1.163809396	-3.513818404
C	-1.414932100	0.136738629	-3.549951800
C	-0.614897270	1.321131576	-3.489907929
C	-1.150509429	2.443901338	-2.795128659
C	-0.293915721	3.270150653	-1.979200373
C	1.112406781	3.042096258	-1.912382552
C	1.753318972	3.300689098	-0.666800316
C	2.910733971	2.532105929	-0.254945529
C	3.449196218	1.518626062	-1.098414538
C	4.104620002	0.403617598	-0.463920598
C	4.124065185	-0.934023332	-1.064830007
C	3.256951189	-1.191222001	-2.195465239
C	2.680192236	-0.058957148	-2.869663220
C	1.414932100	-0.136738629	-3.549951800
C	0.807027337	1.163809396	-3.513818404
C	1.663479361	2.032690859	-2.759313085
C	2.823967151	1.286037727	-2.362445213
Dy	-1.857790973	0.838080687	0.016235936
Dy	1.857790973	-0.838080687	0.016235936
O	0.000000000	0.000000000	0.018572975

References

1. O. V. Dolomanov, L. J. Bourhis, R. J. Gildea, J. A. Howard and H. Puschmann, *Journal of Applied Crystallography*, 2009, **42**, 339.
2. G. M. Sheldrick, *Acta Crystallographica Section C: Structural Chemistry*, 2015, **71**, 3.
3. D. N. Laikov and Y. A. Ustynuk, *Russ. Chem. Bull.*, 2005, **54**, 820.
4. L. Ask Hjorth, M. Jens Jørgen, B. Jakob, E. C. Ivano, C. Rune, D. Marcin, F. Jesper, N. G. Michael, H. Bjørk, H. Cory, D. H. Eric, C. J. Paul, et al., *J. Phys. Condens. Matter*, 2017, **29**, 273002.
5. N. Chen, C. M. Beavers, M. Mulet-Gas, A. Rodriguez-Fortea, E. J. Munoz, Y.-Y. Li, M. M. Olmstead, A. L. Balch, J. M. Poblet and L. Echegoyen, *J. Am. Chem. Soc.*, 2012, **134**, 7851.
6. Y. Feng, T. Wang, J. Wu, L. Feng, J. Xiang, Y. Ma, Z. Zhang, L. Jiang, C. Shu and C. Wang, *Nanoscale*, 2013, **5**, 6704.
7. A. Liu, M. Nie, Y. Hao, Y. Yang, T. Wang, Z. Slanina, H. Cong, L. Feng, C. Wang and F. Uhlik, *Inorg. Chem.*, 2019, **58**, 4774.
8. D. Gatteschi, R. Sessoli and J. Villain, *Molecular Nanomagnets*, Oxford University Press, New York, 2006.
